# *Streptococcus mutans* Secreted Products Inhibit *Candida albicans* Induced Oral Candidiasis

**DOI:** 10.3389/fmicb.2020.01605

**Published:** 2020-07-15

**Authors:** Jéssica Diane dos Santos, Luciana Ruano de Oliveira Fugisaki, Rebeca Previate Medina, Liliana Scorzoni, Mariana de Sá Alves, Patrícia Pimentel de Barros, Felipe Camargo Ribeiro, Beth Burgwyn Fuchs, Eleftherios Mylonakis, Dulce Helena Siqueira Silva, Juliana Campos Junqueira

**Affiliations:** ^1^Department of Biosciences and Oral Diagnosis, Institute of Science and Technology, São Paulo State University (UNESP), São José dos Campos, Brazil; ^2^Department of Organic Chemistry, Institute of Chemistry, São Paulo State University (UNESP), Araraquara, Brazil; ^3^Division of Infectious Diseases, Rhode Island Hospital, Warren Alpert Medical School of Brown University, Providence, RI, United States

**Keywords:** *Streptococcus mutans*, *Candida albicans*, biofilm, filamentation, oral candidiasis

## Abstract

In the oral cavity, *Candida* species form mixed biofilms with *Streptococcus mutans*, a pathogenic bacterium that can secrete *quorum sensing* molecules with antifungal activity. In this study, we extracted and fractioned culture filtrate of *S. mutans*, seeking antifungal agents capable of inhibiting the biofilms, filamentation, and candidiasis by *Candida albicans*. Active *S. mutans* UA159 supernatant filtrate components were extracted via liquid-liquid partition and fractionated on a C-18 silica column to resolve *S. mutans* fraction 1 (SM-F1) and fraction 2 (SM-F2). We found anti-biofilm activity for both SM-F1 and SM-F2 in a dose dependent manner and fungal growth was reduced by 2.59 and 5.98 log for SM-F1 and SM-F2, respectively. The SM-F1 and SM-F2 fractions were also capable of reducing *C. albicans* filamentation, however statistically significant differences were only observed for the SM-F2 (*p* = 0.004). SM-F2 efficacy to inhibit *C. albicans* was confirmed by its capacity to downregulate filamentation genes *CPH1*, *EFG1*, *HWP1*, and *UME6*. Using *Galleria mellonella* as an invertebrate infection model, therapeutic treatment with SM-F2 prolonged larvae survival. Examination of the antifungal capacity was extended to a murine model of oral candidiasis that exhibited a reduction in *C. albicans* colonization (CFU/mL) in the oral cavity when treated with SM-F1 (2.46 log) and SM-F2 (2.34 log) compared to the control (3.25 log). Although both SM-F1 and SM-F2 fractions decreased candidiasis in mice, only SM-F2 exhibited significant quantitative differences compared to the non-treated group for macroscopic lesions, hyphae invasion, tissue lesions, and inflammatory infiltrate. Taken together, these results indicate that the SM-F2 fraction contains antifungal components, providing a promising resource in the discovery of new inhibitors for oral candidiasis.

## Introduction

Oral candidiasis is the most common opportunistic fungal infection of the oral cavity and represents a significant clinical problem, especially in elderly denture wearers, HIV-infected individuals, and patients undergoing antineoplastic therapy (Coronado-Castellote and Jimenez-Soriano, 2013; Garcia-Cuesta et al., 2014; Seneviratne and Rosa, 2016; Zhang et al., 2016; Venkatasalu et al., 2020). The manifestations of oral candidiasis can occur in the lips, skin, and mucosa and have different clinical and histopathological variants (Coronado-Castellote and Jimenez-Soriano, 2013; Costa et al., 2013b; Zhang et al., 2016). Among them, the most common lesions are pseudomembranous candidiasis, characterized by white patches on the oral mucosa surface, and erythematous candidiasis, formed by localized erythema mainly on the tongue and the palate (Costa et al., 2013b). Due to damage to the mucosal surface, patients often complain of dysgeusia and burning sensation that can result in significant patient morbidity through dysphagia, dehydration, and malnutrition (Berberi et al., 2015; Salvatori et al., 2016; Zhang et al., 2016).

The pathogenesis of *Candida* species is associated with various virulence factors, including biofilm formation, morphological transition between yeast and hyphae, and secretion of hydrolytic enzymes (Costa et al., 2013b; Shu et al., 2016). Among them, biofilm formation has gained considerable attention in the last years for the ability to augment *Candida albicans* resistance to antifungal drugs (Costa et al., 2013a; Matsubara et al., 2016). *Candida* biofilm formation initiates through adherence of yeast to host cells or abiotic surfaces of dental prostheses and other medical devices. Once attached, yeasts form colonies, produce germ tubes and hyphae, and secret polysaccharides that contribute to the three-dimensional structure of the biofilm (Costa et al., 2013b). Therefore, the pathogenicity of *C. albicans* involves a significant co-regulation between adherence, biofilm formation, and hyphal development (Breger et al., 2007).

Oral candidiasis is generally managed with the use of azoles antifungal, such as nystatin, fluconazole, ketoconazole, and miconazole (Berberi et al., 2015; Moges et al., 2016; Patton, 2016). However, the combination of frequent recurrences and repeated treatment with antifungal agents leads drug resistant *Candida* strains (Vazquez, 2010; Liu et al., 2015), precipitating the necessity of new therapeutic approaches. One of the possible therapeutic alternatives includes the identification of new antifungal agents from natural substances produced by the human microbiome (Vilela et al., 2015; Barbosa et al., 2016; Rossoni et al., 2018). It is known that bacteria-fungi interactions are common in humans and can influence the transition between health and disease in specific environments within the host (Peleg et al., 2010; Barbosa et al., 2016).

In the oral cavity, *C. albicans* can form mixed biofilms with *Streptococcus mutans*, an important oral bacterium present in the majority of the world population (Becker et al., 2002; Mitchell, 2003). One of the main virulence factors of this bacterium is the ability to form biofilm on enamel surface leading to the development of dental caries in the presence of sucrose (Zhang et al., 2009). In mixed biofilms, *C. albicans* and *S. mutans* can influence each other through cell-cell interactions and via secretion of extracellular signaling molecules (*quorum sensing*) (Pereira-Cenci et al., 2008; Barbosa et al., 2016). The physical interactions between *C. albicans* and *S. mutans* in the presence of sucrose have been associated with a mutualism relationship in which the adherence of both species on dental surfaces are stimulated and can increase the severity of dental caries (Falsetta et al., 2014). However, recent studies reported that the secretion of *quorum sensing* molecules by *S. mutans* can inhibit the filamentation and virulence of *C. albicans* (Pereira-Cenci et al., 2008; Joyner et al., 2010; Barbosa et al., 2016). Genetic studies with different *S. mutans* isolates have shown an enormous diversity of genes related to interspecies communication (Bekal-Si Ali et al., 2002; Klein et al., 2006) and production of a antimicrobial peptide called mutacin (Kamiya et al., 2008).

In our previous study, we evaluated the effects of *S. mutans* (UA159) culture filtrate on *C. albicans* and found it exerted inhibitory activity on biofilm formation, morphogenesis, and pathogenicity of *C. albicans* (Barbosa et al., 2016). The present study extracts, fractionates, and identifies *S. mutans* UA159 culture filtrate specific fraction that inhibits *C. albicans*. *In vitro* and *in vivo* assays are undertaken to understand the mechanisms of action to inhibit biofilm, filamentation, and candidiasis development. For the first time, the effects of *S. mutans* extracts were evaluated on murine oral candidiasis to treat this common oral infection.

## Materials and Methods

### Microorganisms and Culture Conditions

The described assays used *C. albicans* ATCC 18804 and *S. mutans* UA 159 strains from the Oral Microbiology and Immunology Laboratory of the Institute of Science and Technology of São José dos Campos/UNESP. *C. albicans* was grown in agar Sabouraud Dextrose (Himedia Laboratories, Mumbai, India) for 24 h at 37°C and *S. mutans* was cultivated in BHI agar (Himedia, Mumbai, India) for 24 h at 37°C with 5% CO_2_.

### Preparation of *S. mutans* Culture Filtrate

*Streptococcus mutans* was cultured in BHI broth for 24 h at 37°C with 5% CO_2_. After overnight growth, 1 mL of a standardized suspension containing 10^7^ cells/mL was inoculated into 6 mL of BHI broth and incubated for 4 h at 37°C (5% CO_2_) (Barbosa et al., 2016). The culture was centrifuged at 5000 rpm for 10 min and the supernatant was filtered with a 0.22 μm diameter pore membrane using a vacuum filtration system (Stericup^®^ and Steritop^®^ Filter Unit, Millipore, MA, United States).

### Preparation of Crude Extract and Fractions From *S. mutans* Culture Filtrate

The preparations of crude extract and fractions were performed according to National Committee for Clinical Laboratory Standards (2002), Medina et al. (2019) with some modifications. The *S. mutans* culture filtrate was extracted with ethyl acetate (3 × 50% of the supernatant volume) and then the organic solvent was removed under vacuum in a rotary evaporator (Buchi R-114). The obtained crude extract was lyophilized and weighed. Thereafter, the crude extract was fractionated on a C-18 derivatized silica column (150 g, Φ = 3.5 cm) using different MeOH:H_2_O solutions (36:64, 49:51, 60:40, 76:24, 100:00) from which the eluent yielded 5 fractions (360 mL each; SM-F1 to SM-F5). The mass efficiency obtained from SM-CE (1,474 g) for each fraction was: SM-F1 969.3 mg, SM-F2 210.0 mg, SM-F3 22.7 mg, SM-F4 6.0 mg and SM-F5 397.2 mg. Due to the low mass obtained, the fractions SM-F3 and SM-F4 were not included in this study. The fraction SM-F5 was not active on preliminary *in vitro* experiments (data not shown). Therefore, only fractions SM-F1 and SM-F2 were included in this study.

### Minimal Inhibitory Concentration

The antifungal activity was determined by broth microdilution method in accordance with the Clinical and Laboratory Standards Institute (CLSI) document M27-A2 (National Committee for Clinical Laboratory Standards, 2002). For this, *C. albicans* was grown in YPD broth (Himedia, Mumbai, India) and incubated at 30°C for 16 h. The SM-F1 and SM-F2 fractions were diluted in RPMI 1640 medium (Merck, Darmstadt, German) and concentrations from 1 to 15 mg/mL were tested. A suspension of *C. albicans* at 1 × 10^3^ cells/mL was added to each well of 96-well microtiter plates (Kasvi, São José dos Pinhais, Brazil) and incubated at 35°C for 48 h. The results were analyzed after 48 h at 595 nm. The minimal inhibitory concentration (MIC) was considered as the concentration in which no turbidly (growth) was observed.

### Biofilm Formation Assay

The biofilm formation assay was based on the methodologies described by Thein et al. (2006) and Barbosa et al. (2016). For this, 100 μL of 10^7^ cells/mL suspension prepared in Yeast Nitrogen Base broth (YNB, Difco, Detroit, MI, United States) supplemented with 100 mM glucose were added in a 96 well plate and incubated at 37°C at 75 rpm for 90 min. Next, the wells were washed with buffered phosphate saline (PBS, 0.1 M, pH 7.2) two times aiming to remove non-adherent cells. To promote the biofilm formation, YNB supplemented with 100 mM glucose was added and the plates were incubated at 37°C at 75 rpm for 24 h. Then, the media was replaced and fractions SM-F1 and SM-F2 were added. The plates were incubated for 24 h.

After the incubation period, the wells were washed with PBS. A volume of 250 μL of PBS was added to each well and the biofilms were disrupted using an ultrasonic homogenizer (Sonics Vibra Cell) for 30 s at 50 W power setting. Serial dilutions were prepared and 10 μL aliquots of the dilutions were plated on Sabouraud Dextrose agar (Himedia, Mumbai, India). The plates were incubated at 37°C for 48 h and the number of CFU/mL was counted. Two experiments replicates were performed, using five biofilms per group.

### *C. albicans* Filamentation Assay

*Candida albicans* was grown at 37°C for 24 h in YNB supplemented with 100 mM glucose. In a 24 well plate, 100 μL of 10^7^ cells/mL of *C. albicans* were inoculated in 1 mL of sterile distilled water supplemented with 10% fetal bovine serum (Sigma-Aldrich, São Paulo, Brazil) and the fractions SM-F1, SM-F2, or PBS (control group) were added to each well. After incubation at 37°C for 24 h, the pH value was measured and a 50 μL volume was transferred to glass slides, previously demarcated with 10 fields on the back. The slides were then observed under a light microscope (Carl Zeiss, Primo Star, Germany) at 400× magnification (Barbosa et al., 2016). To quantify the hyphae formation, 10 fields were analyzed in each slide, totaling 100 fields for each sample. A score was assigned according to the number of hyphae presence: 0, absence of hyphae; 1, from 1 to 10; 2, from 11 to 20; 3 from 21 to 30; 4, from 31 to 40; 5, more than 40. Three (SM-F1) and five (SM-F2) assays were used per group and the experiment was performed as independent duplicates. Next, the study of *C. albicans* filamentation was complemented with analysis of scanning electron microscopy and gene expression.

### Analysis of Filamentation by Scanning Electron Microscopy (SEM)

Acrylic resin disks measuring 5 mm in diameter and 3 mm in thickness were deposited at the bottom of each well in the 24 well plates for filamentation induction. After 24 h of incubation, the disks were removed from the wells and washed with 1 mL of 2.5% glutaraldehyde (Sigma-Aldrich, São Paulo, Brazil) for 1 h at 37°C. The disks were removed from glutaraldehyde and dehydrated with increasing ethanol washes (10, 25, 50, 75, and 90%) for 20 min each, followed by immersion in absolute alcohol for 1 h. They were then transferred to aluminum stubs and analyzed using an Inspect S50 scanning electron microscope (FEI, Czechia). The assay was performed in duplicate with three resin disks per group.

### Analysis of Gene Expression Related to Filamentation of *C. albicans*

Five genes associated with *C. albicans* filamentation were evaluated: *CPH1*, *EFG1*, *HWP1*, *UME6*, and *YWP1*. This experiment was performed in 24 well plates using the same conditions and fraction treatments described in section “*C. albicans* Filamentation Assay.” After the incubation period, *C. albicans* was collected by centrifugation at 4700 rpm for 5 min and RNA extraction was performed using TRIzol, following the manufacturer’s suggested protocol (Ambion, Inc., Carlsbad, CA, United States).

The concentration and purity of the RNA was determined using a NanoDrop 2000 spectrophotometer (Thermo Fisher Scientific Inc., Wilmington, DE, United States) by measuring the absorbance at optical densities (OD): 230, 260, and 280 nm. The OD260/OD280 ranged from 1.80 to 2.00 and OD260/OD230 ranged from 2.00 to 2.10. The integrity of the RNA was further checked in a selected subset of samples by electrophoresis through 1% non-denaturing agarose gels.

The extracted total RNA (720 ng) was treated with DNase I (Turbo DNase Treatment and Removal Reagents - Ambion Inc., Carlsbad, CA, United States) and transcribed into complementary DNA (cDNA) using the SuperScript III First-Strand Synthesis SuperMix for qRT-PCR Kit (Invitrogen, Carlsbad, CA, United States), according to the protocols recommended by the manufacturer. All investigated samples were transcribed with the same reverse transcription reaction conditions. Negative controls, which were run simultaneously and used in all experiments, did not contain either RNA (no template control) or no reverse transcriptase (RT negative control), that could indicated RNA and genomic DNA contamination, respectively. Primers for the genes analyzed are listed in Table 1.

**TABLE 1:** Quantitative real-time PCR primers.

Target gene	Oligonucleotide sequence 5′-3′*	Description	Amplicon size (bp)**	References
*ACT1*	**F**- GAAGCCCAATCCAAAAGA **R**- CTTCTGGAGCAACTCTCAATTC	Structural constituent of cytoskeleton (Normalizing internal standard)	130 bp	Nailis et al., 2006
*RPP2B*	**F**-TGCTTACTTATTGTTAGTTCAAGGTGGGTA **R**- CAACACCAACGGATTCCAATAAA	Structural constituent of ribosome (Normalizing internal standard)	83 bp	Nailis et al., 2006
*PMA1*	**F**- TTGCTTATGATAATGCTCCATACGA **R**- TACCCCACAATCTTGGCAAGT	Plasma Membrane H (+) ATPase (Normalizing internal standard)	66 bp	Nailis et al., 2006
*CPH1*	**F**- ACGCAGCCACAAGCTCTACT **R**- GTTGTGTGTGGAGGTTGCAC	*Candida* pseudohyphal regulator (transcription factor activity)	119 bp	Sherry et al., 2014
*EFG1*	**F**- CAGTATGGTCAGTATAATGCT **R**- TGTTGTTGCTGTTGGTATGGATATGATGATG	Enhanced filamentous growth (transcription factor activity)	222 bp	Hnisz et al., 2012
*HWP1*	**F**- GAAACCTCACCAATTGCTCCAG **R**- GTAGAGACGACAGCACTAGATTCC	Hyphal wall protein (cell adhesion molecule binding)	92 bp	Hnisz et al., 2012
*UME6*	**F**- TCATTCAATCCTACTCGTCCACC **R**- CCAGATCCAGTAGCAGTGCTG	Zn(II)2Cys6 transcription factor (transcription factor activity)	133 bp	Hnisz et al., 2012
*YWP1*	**F**- ACACCGGAAAATACCGTTGC **R**- ATGGCAGCTTTACCAGAACC	Yeast-form wall protein (adhesion of symbiont to host)	116 bp	Granger, 2012

*F- Forward and R- Reverse; ** bp- base pairs.

For real-time quantitative PCR (qPCR) reactions, the Platinum SYBR Green qPCR SuperMix-UDG Kit (Invitrogen, Carlsbad, CA, United States) was used as recommended by the manufacturer. The reactions were performed in triplicate in the StepOnePlus Real-Time PCR System (Applied Biosystems, Foster, CA, United States), consisting of 12.5 μL SuperMix Platinum SYBR Green, 1 μL ROX, 0.3 μM each for specific forward and reverse primers (final concentration), and target cDNA, supplemented with RNAse-free ddH_2_O to a final volume of 25 μL. Cycling parameters for amplification reactions were 50°C for 2 min; 95°C for 2 min; followed by 40 cycles of 95°C for 15 s and 60°C for 30 s, with dissociation (a melting curve) during the last cycle of 95°C for 15 s, 60°C for 30 s, 95°C for 15 s. All samples showed only a single peak, indicating a single pure product and no primer/dimer formation. Real-time PCR efficiencies were acquired by amplification of standardized serial dilutions of the template cDNA and were determined for each gene as the slope of a linear regression model (Supplementary Figure S1). The 2^–ΔΔ*CT*^ method was used to analyze the relative changes in gene expression from the quantitative RT-PCR experiment (Livak and Schmittgen, 2001).

The transcribed cDNAs were amplified for relative quantification of the expression of the *CPH1*, *EFG1*, *HWP1*, *UME6*, and *YWP1* genes in relation to the concentration of the reference gene *PMA1*. In this study, three reference genes, *ACT1*, *PMA1*, and *RPP2B*, were tested in all experimental groups. The obtained results were analyzed and the selected reference gene was *PMA1* (Supplementary Figure S2).

### *In vivo* Study in *G. mellonella* Model

*Galleria mellonella* larvae were used in the final larval stage weighting from 250 to 300 mg according to methodologies previously established by our group (Barbosa et al., 2016; Rossoni et al., 2017). Initially, the *S. mutans* extract in various concentrations was injected in healthy larvae to evaluate its toxicity. To study *S. mutans* fractions effects on candidiasis, larvae were infected with 10^8^ cells/mL *C. albicans* containing, and after 30 min, treated with an injection of the SM-F1 or SM-F2 fractions. Untouched larvae and PBS injected larvae were used as control groups. After injections, the larvae were stored in petri dishes at 37°C. *G. mellonella* survival were monitored daily for 7 days. Larvae were considered dead when they failed to react to touch. The death of all larvae in the experimental group or the transition to pupa determined the end of the experiment. Sixteen larvae were used in each group.

### *In vivo* Murine Oral Candidiasis Model

Adult male *Swiss* mice, weighing approximately 30 g, from the Central Animal Care Facility of UNESP (Botucatu, SP, Brazil) were used in the study with approval from the Ethics Committee on the Use of Animals of the ICT/UNESP under protocol 005/2016-CEUA- ICT-UNESP. A total of 17 mice were used, divided into the following groups: mice not infected by *C. albicans* and not treated (*n* = 2), mice infected by *C. albicans* and not treated (*n* = 5), mice infected by *C. albicans* and treated with SM-F1 (*n* = 5), and mice infected by *C. albicans* and treated with SM-F2 (*n* = 5).

Oral candidiasis was induced as previously described (Takakura et al., 2003). Animals were immunosuppressed with two intraperitoneal injections of 100 mg/kg prednisolone (Depo-Medrol, Laboratórios Pfizer Ltda., Guarulhos, Brazil) on days alternating with *Candida* inoculations. Tetracycline hydrochloride (Terramycin, Pfizer) was administered at a concentration of 0.83 mg/mL in the drinking water during the experiment period. For *C. albicans* infection, animals were sedated intraperitoneally by injections of ketamine (100 mg/kg) and xylazine (10 mg/kg) and inoculated with a *C. albicans* suspension (10^9^ cells/mL) using a sterile swab. After 24 h of the second infection, 200 μL of the SM-F1 or SM-F2 at the concentration of 1 g/Kg were delivered into the oral cavity using a pipette.

### Recovery of *C. albicans* From the Oral Cavity After Treatments

After 48 h of treatment, samples were collected from the tongue dorsum with a swab, placed in a tube containing 0.9 mL of PBS, and shaken for 1 min. Considering that the swab absorbed approximately 0.1 mL of saliva from the oral cavity of mice, this solution was estimated to be a 10^–1^ starting dilution of *Candida* from the soaked swab. Serial dilutions were performed and seeded on Sabouraud dextrose agar (Difco, Detroit, United States) supplemented with 0.1 mg/mL of chloramphenicol (Vixmicina, São Paulo, Brazil). The plates were incubated at 37°C for 48 h to determine the number of CFU/mL. Immediately after sample collections, mice were euthanized by administering an overdose of anesthetic and the tongues were removed for macroscopic and microscopic analysis.

### Macroscopic Analysis of Oral Candidiasis

Characteristics lesions of candidiasis on the dorsum tongue were analyzed and quantified using a stereomicroscope (Zeiss, Göttingen, Germany). Scores of 0–4 were assigned according to the extension of lesions: 0, absence of lesions (normal aspect of tongue); (1) white plaques in less than 20%; (2) white plaques ranging from 21 to 90%; (3) white plaques in more than 91%; (4) thick white plaque as pseudomembranes in more than 91% (Takakura et al., 2003; Rossoni et al., 2015).

### Microscopic Analysis of Oral Candidiasis

For the microscopic analysis, the tongues were fixed in 10% formalin for 24 h and sectioned in two parts in the sagittal direction. After being imbedded in paraffin, serial 5 μm thick sections were obtained, and then Hematoxylin-Eosin (HE) and Schiff’s Periodic Acid (PAS) stained (Rossoni et al., 2015). The presence of candidiasis was investigated on the entire surface of the dorsum tongue and the description of the histological sections was carried out according to the presence of yeasts and hyphae, extent of lesions and changes in the tissues involved.

The presence of yeasts and hyphae was quantified according to the methodology described by Junqueira et al. (2005), attributing the following scores to the histological fields: 0, absence of yeasts/hyphae; 1, 1 to 5 yeasts/hyphae; 2, 6 to 15 yeasts/hyphae; 3, 16 to 50 yeasts/hyphae; 4, more than 50 yeasts/hyphae. The intensity of tissue lesions and inflammatory response in connective tissue was made according to Martins Jda et al. (2011). The tissue lesions were evaluated by the presence of epithelial hyperplasia, disorganization of the basal layer, exocytosis, spongiosis, loss filiform papillae, hyperkeratosis, desquamation, acantholysis, loss of stratification and formation of intraepithelial micro abscesses. For the chronic inflammatory infiltrate, the following scores were assigned: 0 (absence of inflammatory cells), 1 (mild inflammatory infiltrate), 2 (moderate inflammatory infiltrate) and 3 (severe inflammatory infiltrate).

### Analysis of *S. mutans* Supernatant Fractions by Gas Chromatography

The fractions SM-F1 and SM-F2 were analyzed by gas chromatography coupled to mass spectrometry (GC-MS, Shimadzu, model GC-MS-QP2020, with auto-injector AOC-20i), using a Supelco RTX-5MS column (5% phenyl polymethylsiloxane; 30 m × 0.25 mm × 0.25 μm) (Pellati and Benvenuti, 2007). Chromatographic conditions were: injector temperature −260°C, injection mode - split; detector temperature −260°C; carrier gas flow (He) −1.0 mL min^–1^; using the following temperature program −80°C with a 3 min hold, ramped to 260°C at 3°C/min and held for 10 min. The mass spectrometer used in the CG-MS analyzes is equipped with an electronic impact ionization (EI) source, set to 70 eV and a mass range of 35–700 *m/z* acquisition. To ensure greater thermal stability and greater volatility of the constituents, as well as better chromatographic resolution (Pellati and Benvenuti, 2007), a derivation step (silylation) was performed during sample preparation. Thus, 5 mg of SM-F1 and SM-F2 fraction were solubilized in 200 μL of pyridine, then 200 μL of N-methyl-N- (trimethylsilyl) - trifluoroacetamide (MSTFA - Sigma) were added.

To compare the GC-MS results obtained from SM-F1 and SM-F2, BHI broth was prepared and extracted with ethyl acetate (3 × 50% of its volume). After the removal of the organic solvent using a rotary evaporator, the obtained material (BHI control) was prepared, including a silylation step as conducted for fractions SM-F1 and SM-F2, to be analyzed by GC-MS in the same conditions described above. The identification of the compounds was carried by the comparison of mass spectra, obtained for each peak during analyses, with data available in Wiley7 and NIST libraries using GCMSsolutions Version 2.5 software. Only compounds that presented similarity higher than 85% were described.

### Statistical Analysis

GraphPad Prism 8.4.2 statistical software (San Diego, United States) was used in all tests with a *p* ≤ 0.05 considered significant. The results of biofilms (CFU/mL) and gene expression were evaluated by the Student’s *t*-test. Kruskal-Wallis and Dunn tests were used to compare the scores obtained in the *in vitro* filamentation assay, macroscopic and microscopic analysis in mice. The number of *C. albicans* CFU/mL recovered from the oral cavity of mice were submitted to ANOVA and Tukey test. For the survival experiments in *G. mellonella*, the survival curve was generated using the Kaplan-Meier method and the level of significance calculated by the Log-rank test (Mantel-Cox).

## Results

### Antifungal and Anti-biofilm Activities of SM-F1 and SM-F2

**FIGURE 1:**
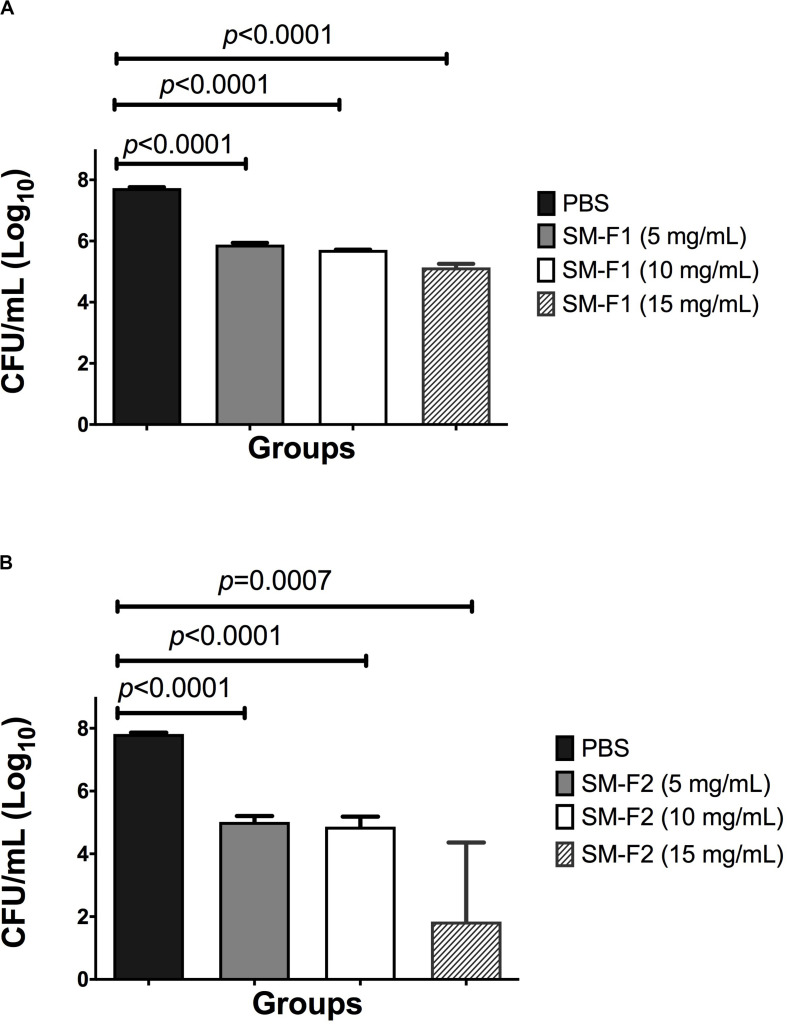
Analysis of *in vitro Candida* biofilms. Mean and SD of CFU/mL (Log_10_) of *C. albicans* viable cells obtained for the non-treated control group (PBS) and the experimental groups treated with the SM-F1 **(A)** or SM-F2 **(B)** fractions at 5, 10, and 15 mg/mL. Student’s *t*-test was used to compare the control group with the experimental groups.

Initially, the antifungal activity of SM-F1 and SM-F2 fractions were tested on planktonic growth of *C. albicans*. SM-F2 fraction was active against *C. albicans* with MIC of 15 mg/mL. However, no antifungal activity on planktonic cells was observed for the SM-F1 fraction. After that, the fractions were tested against *C. albicans* biofilms, using concentrations of 5, 10, and 15 mg/mL for both SM-F1 and SM-F2. We observed reductions in the CFU/mL number of *C. albicans* for the biofilms treated with SM-F1 and SM-F2 in all tested concentrations when compared to the non-treated control group. The biofilms treated with SM-F1 at 5, 10 and 15 mg/mL showed reductions of 1.85, 2.02, and 2.59 logs, respectively. A more substantial reduction was found when biofilms were treated with SM-F2, reaching 2.8 for 5 mg/mL SM-F2, 2.95 for 10 mg/mL SM-F2 and 5.98 logs with 15 mg/mL SM-F2. In the group treated with 15 mg/mL SM-F2 (*n* = 5), three biofilms showed a total inhibition of *C. albicans* cells (Figure 1). Therefore, SM-F1 and SM-F2 exhibited anti-biofilm activity against *C. albicans* in a dose dependent manner, with SM-F2 being the most effective inhibitory fraction. Further, SM-F2 had conserved activity against planktonic cell forms.

### Activity of SM-F1 and SM-F2 on *Candida* Filamentation

**FIGURE 2:**
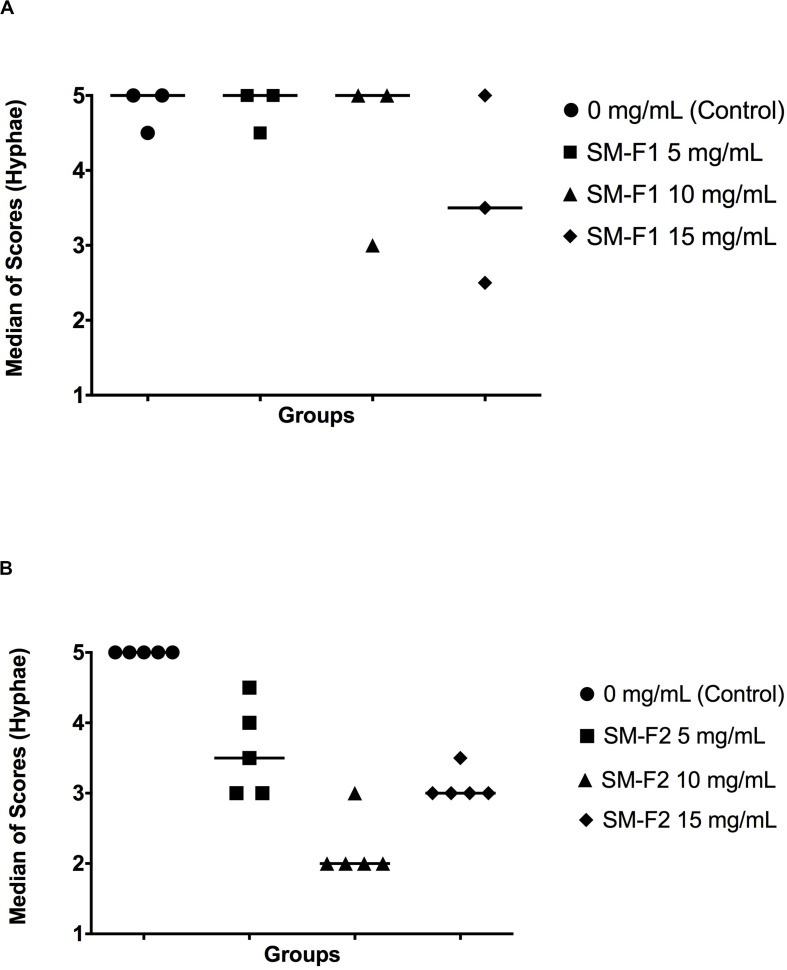
Quantitative analysis of *Candida* filamentation. Median scores obtained by counting hyphae counting. Scores were assigned according to the number of hyphae in each microscopic field. **(A)** *C. albicans* cultured with PBS (control group), SM-F1 5 mg/mL, SM-F1 10 mg/mL, and SM-F1 15 mg/mL. No statistically significant differences were found between groups. **(B)** *C. albicans* cultured with PBS (control group), SM-F2 5 mg/mL, SM-F2 10 mg/mL, and SM-F2 15 mg/mL. Statistically significant difference was observed only between the control group and SM-F2 10 mg/mL (*p* = 0.004). Kruskal-Wallis and Dunn’s test.

In the microscopic analysis of filamentation, the group treated with SM-F1 faction at 5 mg/mL exhibited numerous hyphae spread along the microscopic fields similar to the profile observed in the non-treated control group (Supplementary Figure S3). The SM-F1 fraction of 10 and 15 mg/mL led to a discrete reduction in the quantity of hyphae formation without statistically significant difference in relation to the control group. However, when *C. albicans* was cultured with the SM-F2 fraction, a hyphae reduction was found for all tested concentrations (5, 10, and 15 mg/mL). Statistically significant differences in relation to the control group were observed only for the groups treated with 10 mg/mL SM-F2 (Figure 2).

Since it is known that the acidic pH may have influence on the *C. albicans* filamentation, the pH value of medium was measured before the microscopic analysis. For all groups, we found pH values from 6.8 to 7.0, indicating that the pH of the medium did not interfere with the hyphae inhibition caused by the treatment with *S. mutans* fractions (Supplementary Figure S4).

**FIGURE 3:**
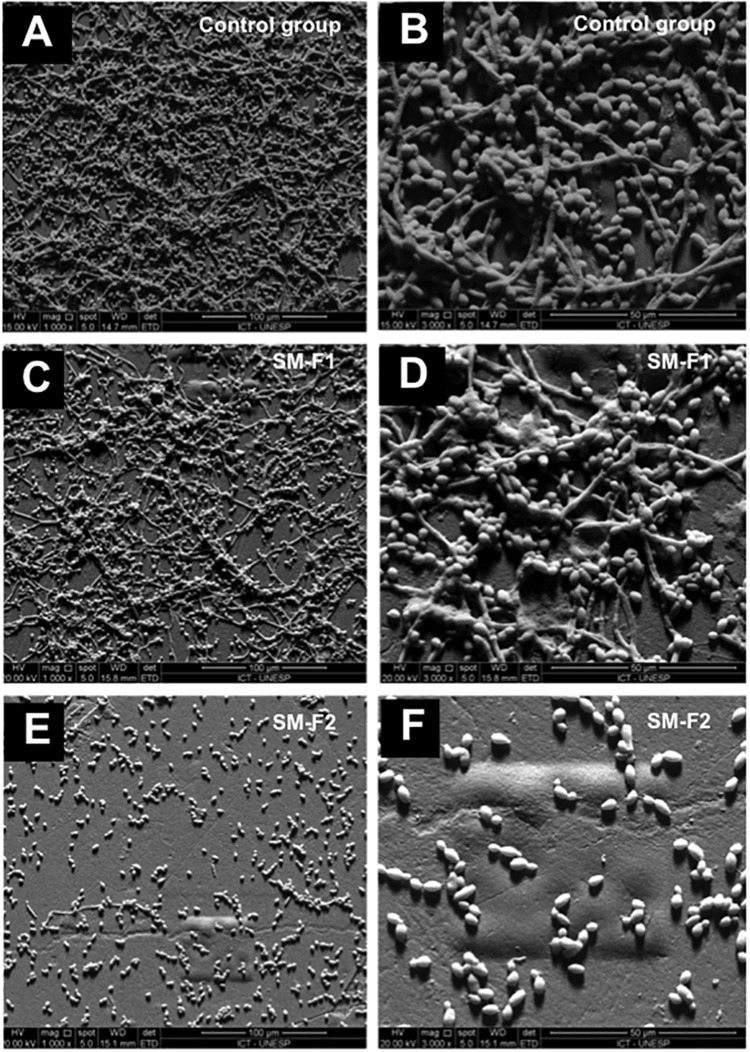
SEM of fungal filamentation. Presence of *C. albicans* yeast and hyphae was assessed within the following groups: non-treated control at 1000× magnification **(A)** and 3000× magnification **(B)**, cells treated with 10 mg/mL SM-F1 at 1000× magnification **(C)** and 3000× magnification **(D)**, and cells treated with 10 mg/mL SM-F2 at 1000× magnifications **(E)** and 3000× magnification **(F)**.

The results of light microscopy analysis were confirmed by the SEM analysis, in which the non-treated control group showed a large number of yeasts and hyphae that were reduced after the treatments with *S. mutans* fractions. Treatment with SM-F2 was more effective in decreasing the quantity of yeast and hyphae in relation to SM-F1. In addition to reducing the quantity of *C. albicans* cells, the SM-F2 fraction exhibited a strong capacity for inhibiting hyphae formation (Figure 3).

### Effects of SM-F2 on Gene Expression of *C. albicans*

**FIGURE 4:**
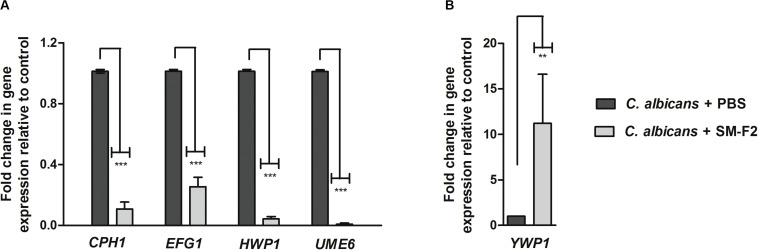
Relative expression of *C. albicans* genes. Relative quantification of *CPH1*, *EFG1*, *HWP1*, and *UME6* genes **(A)** and *YWP1* gene **(B)** for the non-treated control group (PBS) and treated group with SM-F2 (10 mg/mL). Each gene was normalized using the reference gene *PMA1.* Values were expressed as the mean and SD. The Student’s *t*-test was used to compare gene expression between the control and treated group (**p < 0.01 and *** *p* < 0.0001).

To investigate the inhibitory mechanisms of SM-F2 on *C. albicans* filamentation, we analyzed the expressions of five virulence genes: *CPH1*, *EFG1*, *UME6, HWP1*, and *YWP1. CPH1*, *EFG1*, and *UME6* are transcriptional regulators involved in morphogenesis (Gulati and Nobile, 2016). *HWP1* encodes a cell wall protein essential for hyphae development (de Barros et al., 2017), while *YWP1* encodes a cell wall glycoprotein present in the yeast but absent in the filamentous form (Granger, 2012). Most of the evaluated genes were downregulated when *C. albicans* cells were exposed to SM-F2, with reductions of 10.0-, 4.0-, 23.25-, and 111.1-fold for *CPH1, EFG1*, *HWP1*, and *UME6* genes, respectively (*p* < 0.0001) (Figure 4A). Conversely, *YWP1* was upregulated with SM-F2 treatment, increasing expression by 11.22-fold (*p* = 0.0092) (Figure 4B). Taken together, these results reinforce the inhibitory activity of SM-F2 observed *in vitro* filamentation assays and suggest repression of the signaling cascade that leads to filamentation of *C. albicans*.

### Antifungal Activity of SM-F1 and SM-F2 in *G. mellonella* Model

**FIGURE 5:**
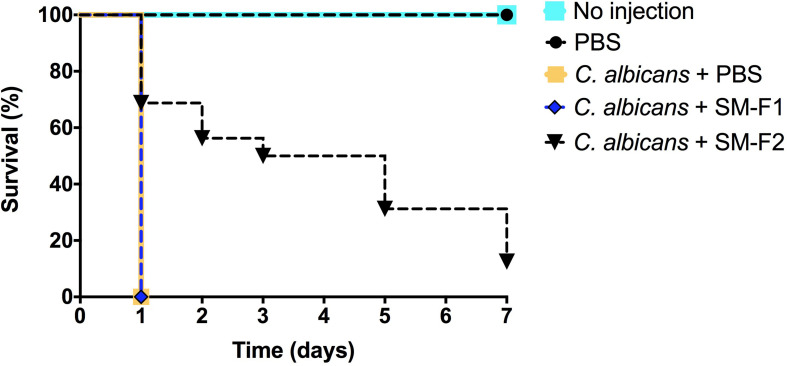
*G. mellonella* survival assays. Antifungal activity of SM-F1 (15 mg/mL) and (SM-F2 15 mg/mL) on larvae infected by *C. albicans*. Statistically significant difference was observed between the group treated with SM-F2 and non-treated control group (PBS) (*p* < 0,0001). Log-rank test (Mantel-Cox).

*Streptococcus mutans* supernatant fractions ranging between 1 and 15 mg/mL were injected into healthy larvae to evaluate toxicity. None of the tested concentrations interfered with larva survival (data not shown). Based on the results obtained in the *in vitro* assays, 15 mg/mL was selected for larvae therapeutic treatment upon *C. albicans* infection. The treatment with SM-F1 did not prolong larvae survival and resulted in 100% of death after 1 day, similar to the non-treated control group. By contrast, treatment with SM-F2 achieved significant larvae survival, with 70% of the larvae surviving beyond 1 day. Although SM-F2 treatment delayed *G. mellonella* death, only 12.5% of the larvae survived until the end of the experiment (7 days) (Figure 5).

### Effects of SM-F1 and SM-F2 on Oral Candidiasis in Mice

**FIGURE 6:**
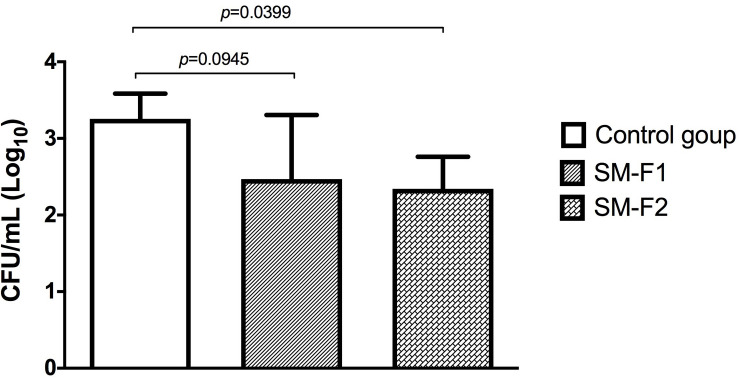
Quantification of *Candida* cells recovered from the oral cavity of mice. Mean and SD of the CFU/mL (Log) of *C. albicans* obtained in the non-treated control group (PBS) and the experimental groups treated with the SM-F1 or SM-F2 fractions (1 g/Kg). ANOVA and Tukey test.

*C. albicans* can cause oral infections that are difficult to treat due to biofilm formation. With the encouraging data that SM-F2 reduces biofilm formation and prolongs *G*. *mellonella* survival, the fractions were evaluated in a murine oral candidiasis model. *C. albicans* cells were recovered from the oral cavity of all infected groups. The CFU/mL counts were 3.25 ± 0.32 (Log_10_) for the non-treated control group, 2.46 ± 0.83 (Log_10_) for the group treated with SM-F1 and 2.34 ± 0.41 (Log_10_) for the group treated with SM-F2. Although both treatments were capable of reducing *C. albicans* colonization, statistically significant difference in relation to the control group was only observed for SM-F2 (Figure 6).

**FIGURE 7:**
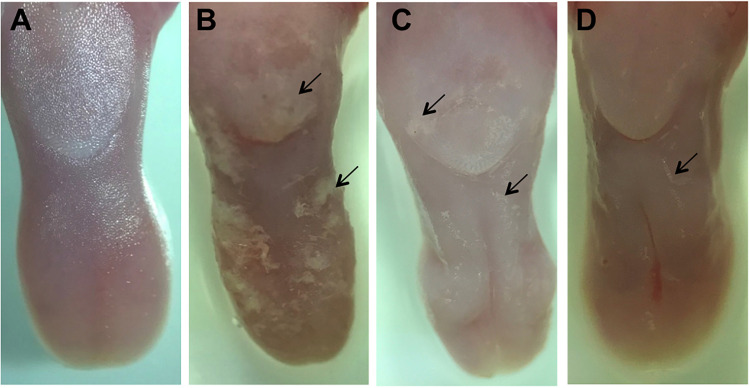
Macroscopic images of the dorsum tongue. Control group not infected by *C. albicans*: normal aspect of tongue **(A)**. Control group infected by *C. albicans* and not treated: presence of numerous white patches of candidiasis lesions (→) and papillary atrophy **(B)**. Groups treated with SM-F1 **(C)** or SM-F2 **(D)** showing few areas of white patches (→) and papillary atrophy.

**FIGURE 8:**
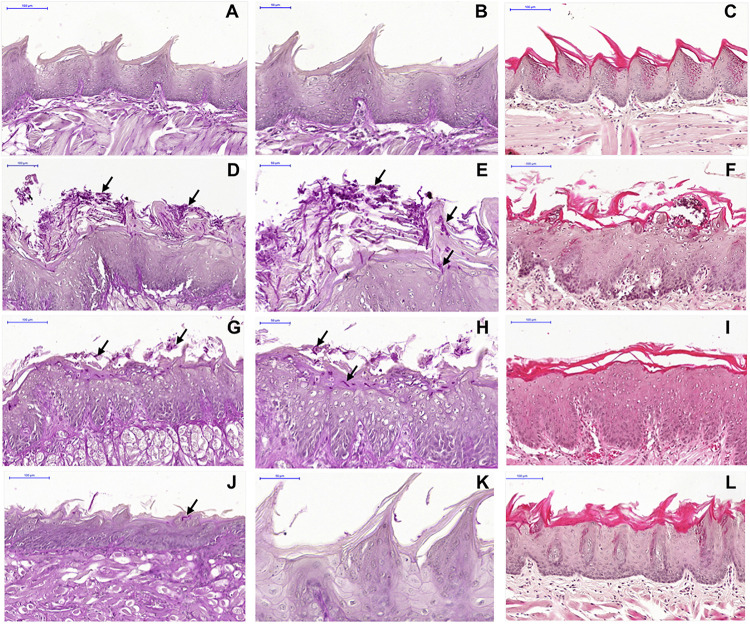
Microscopic images of the dorsum tongue. Control group not infected by *C. albicans* stained by PAS **(A,B)** and H&E **(C)**: normal histological aspect of tongue. Control group infected by *C. albicans* and not treated stained by PAS **(D,E)** and H&E **(F)**: presence of several yeast and hyphae (→) in the keratin layer; epithelial changes with microabscesses, exocytosis, spongiosis and loss of filiform papillae; and formation of inflammatory infiltrate in lamina propria. Groups treated with SM-F1 stained by PAS **(G,H)** and H&E **(I)** or with SM-F2 stained by PAS **(J,K)** and H&E **(L)**: reduction in candidiasis lesions in relation to the not treated control group.

Macroscopic analysis of the dorsum tongue revealed extensive candidiasis lesions in the non-treated control group, characterized by whitish regions with the presence of pseudomembrane and areas of papillary atrophy. Promisingly, these lesions were significantly reduced in the groups treated with SM-F1 and SM-F2. Although, the tongues of treated animals showed papillary atrophy, few whitish lesions characteristic of candidiasis were found in these groups (Figure 7). These findings were confirmed in the microscopic analysis, in which the non-treated control group presented a large quantity of yeasts and hyphae in the keratin layer. In addition, numerous epithelial lesions (microabscesses, exocytosis, spongiosis, and loss of filiform papillae) and intense inflammatory infiltrate were found (Figure 8). Analyzing all the extension of dorsum tongue, we verified that tissue lesions were limited to the areas with the presence of yeast and hyphae. Therefore, the tested concentration exhibited *C. albicans* inhibition without adversely damaging tongue tissue at the tested concentrations.

**FIGURE 9:**
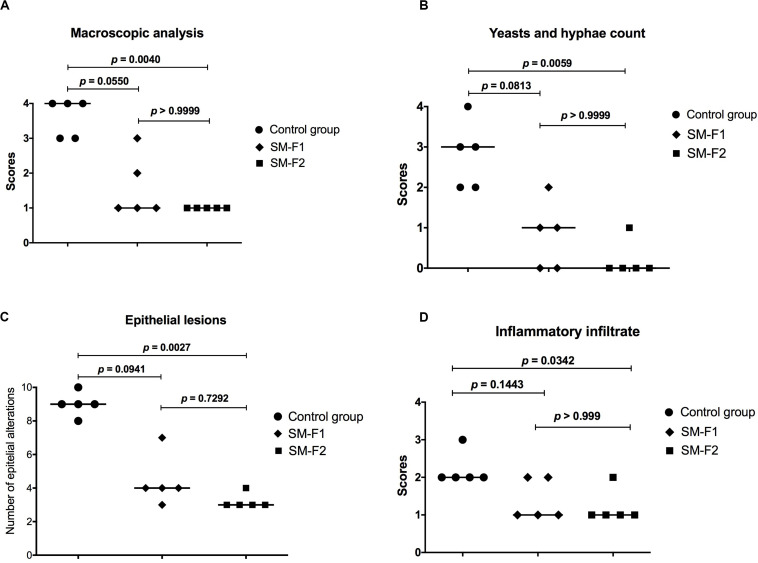
Quantitative analysis of candidiasis lesions formed on the dorsum tongue of mice infected with *C. albicans.* **(A)** Scores and medians obtained in the quantification of macroscopic lesions. **(B)** Scores and medians obtained in yeasts/hyphae counts in the histological cuts stained by PAS. **(C)** Medians of epithelial lesions determined in the histological cuts stained by H&E. **(D)** Scores and medians of infiltrate inflammatory intensity in the histological cuts stained by H&E. Kruskal-Wallis and Dunn’s test.

The macroscopic and microscopic lesions were quantified to compare the studied groups. In the macroscopic analysis, we observed a media of 3.6 ± 0.54 for the non-treated control group, 1.60 ± 0.89 for the group treated with SM-F1 and 1.00 ± 0.00 for the group treated with SM-F2. The similar reduction proportions were found in the microscopic analysis. The medians of scores assigned for yeast/hyphae counts were 3 for the non-treated control group, 1 for the group treated with SM-F1 and 0 for the group treated with SM-F2. Interestingly, the SM-F2 showed a median score equal to 0 that corresponded to total absence of yeasts/hyphae. The number of epithelial lesions and intensity of inflammatory filtrate were also lower in the groups treated with SM-F1 and SM-F2 when compared to non-treated control group. In all the analysis, both fractions decreased the oral candidiasis, however only the SM-F2 fraction showed statistically significant difference in relation to the control group (Figure 9). Taken together, these results suggest that SM-F2 was the most efficient *S. mutans* fraction to treat the oral candidiasis in mice at the present purification and concentration levels.

### Identification of Substances in the SM-F1 and SM-F2 Fractions

The GC-MS analysis of SM-F1 and SM-F2 fractions were performed along with GC-MS analysis of BHI control group in order to identify compounds present in the fractions but absent in the culture medium. Comparison of chromatograms between the SM-F1 fraction and BHI control indicated the presence of peaks in SM-F1 (*t*_*R*_ = 53.12, 55.17, 55.46, 55.84, 56.23, 58.10 min) different from those observed in the BHI control group (Supplementary Figure S5). The comparison between the chromatograms of SM-F2 fraction and BHI control (Supplementary Figure S6) showed some peaks with different intensities from the peaks in the BHI control, including a peak observed only in SM-F2 chromatogram (*t*_*R*_ = 56.41 min).

Despite the great number of peaks found in chromatograms, few compounds were identified due to the low similarity with the substances described in the databases. Since a limited numbers of microbial substances have been identified by GC-MS and inserted in these databases, we could not identify all the compounds presents in the SM-F1 and SM-F2 fractions. Moreover, there is a possibility that some of these substances are not yet known.

The identified substances in the SM-F1 and SM-F2 fractions that showed similarity higher than 85% compared with data from software libraries are described in the Tables 2, 3, respectively. Some of the compounds identified in the SM-F1 fraction were aromatic acids, such as benzoic and benzeneacetic acids (*t*_*R*_ = 13.92 and 16.10 min, respectively), while some of the compounds identified in the SM-F2 fraction were linear carbon chain acids, including hexadecanoic and octadecanoic acids (*t*_*R*_ = 44.30 and 50.33 min, respectively).

**TABLE 2:** Identified compounds by GC-MS presented in *S. mutans* fraction (SM-F1).

Peak#	t_*R*_ (min)	Compounds	Similarity
3	7.15	Propanoic acid, 2-[(trimethylsilyl) oxy]-, trimethylsilyl ester	94%
5	13.92	Benzoic acid, trimethylsilyl ester	93%
6	15.63	Glycerol-tri- trimethylsilyl ether	93%
7	16.10	Benzeneacetic acid, trimethylsilyl ester	98%
**9^a^**	**18.06**	**Propanoic acid, 2,3-bis[(trimethylsilyl) oxy]-, trimethylsilyl ester**	**94%**
10	18.14	Pyrimidine, 2,4-bis[(trimethylsilyl)oxy]-	92%
**12^a^**	**20.13**	**(3R)-3-Methyl-1,4-bis(trimethylsilyl)piperazine-2,5-dione**	**93%**
13	20.49	Silanamine, 1,1,1-trimethyl-N-(trimethylsilyl)-N-[2-[(trimethylsilyl)oxy]ethyl]-	89%
**14 ^a^**	**20.85**	**Pyrimidine, 5-methyl-2,4-bis[(trimethylsilyl)oxy]-**	**88%**
15	21.10	Benzenepropanoic acid, trimethylsilyl ester	95%
30	27.60	4-Hydroxyphenylethanol, di-TMS^b^	92%
55	39.97	Pyridine, 2-methyl-3-(trimethylsilyloxy)-4,5-bis-[(trimethylsilyloxy)methyl]-	94%
56	40.42	Pyrrolo[1,2-a]pyrazine-1,4-dione, hexahydro-3-(2-methylpropyl)-	90%
**59 ^a^**	**41.50**	**Gulose, 2,3,4,5,6-pentakis-O-(trimethylsilyl)-**	**89%**
62	44.24	9H-Purine, 9-(trimethylsilyl)-2,6-bis[(trimethylsilyl)oxy]-	90%
83	54.12	Pyrrolo[1,2-a]pyrazine-1,4-dione, hexahydro-3-(phenylmethyl)-	90%

*^*a*^Compound presented only in SM-F1 when compared with BHI control group. ^*b*^TMS - trimethylsilyl group.*

**TABLE 3:** Identified compounds by GC-MS presented in *S. mutans* fraction (SM-F2).

Peak#	t_*R*_ (min)	Compound	Similarity
5	7.12	Propanoic acid, 2-[(trimethylsilyl) oxy]-, trimethylsilyl ester	94%
6	13.08	Silane, trimethyl(2-phenylethoxy)-	93%
**7^a^**	**14.88**	**Nicotinic acid-TMS^b^**	**92%**
8	15.59	Glycerol-tri-tms ether	93%
41	40.35	Pyrrolo[1,2-a]pyrazine-1,4-dione, hexahydro-3-(2-methylpropyl)-	89%
54	44.30	Hexadecanoic acid, trimethylsilyl ester	88%
66	50.33	Octadecanoic acid, trimethylsilyl ester	86%
**71^a^**	**52.57**	**Tryptophan, 2TMS^b^**	**86%**
73	53.71	9-Octadecenamide, (Z)-	93%
74	54.30	Pyrrolo[1,2-a]pyrazine-1,4-dione, hexahydro-3-(phenylmethyl)-	91%

*^*a*^Compound presented only in SM-F2 when compared with BHI control. ^*b*^TMS - trimethylsilyl group.*

## Discussion

In an effort to seek new antifungals to treat oral candidiasis, we explored the ecological interactions established by bacteria and fungal in the oral microbiome. Among these interactions, *quorum sensing* molecules play an important role to control the oral microbial population and to manipulate the phenotypes of competing species (Vilchez et al., 2010). Since *C. albicans* are found together with *S. mutans* in dental biofilms, we selected this bacterium to investigate the presence of signaling molecules diffuse into the medium with potential antifungal activities (Vilchez et al., 2010; Barbosa et al., 2016). The presence of competence-stimulating peptides (CSP) (Khan et al., 2016) and universal bacterial signal autoinducer-2 (AI-2) was already confirmed in the cell-free culture filtrate of the *S. mutans* UA159 strain (Vilchez et al., 2010). Based on its well-studied *quorum sensing* system, this strain was employed in our study. Analyzing the free-cell culture filtrate of *S. mutans* UA159 using extraction and fractionation methods, we identified 2 bioactive fractions against *C. albicans* (SM-F1 and SM-F2). Both SM-F1 and SM-F2 were examined for inhibitory activity against planktonic cells, only SM-F2 demonstrating efficacy.

These fractions were then tested on biofilms and filamentation of *C. albicans* that are important virulence factors for the candidiasis development. It is know that antifungal agents with the ability to modulate biofilm formation and suppress dimorphic switching can lead the homeostatic balance of oral microbiome, protecting the host from the pathogenicity of *Candida* species (Chanda et al., 2017). Interestingly, fractions SM-F1, and even more so, SM-F2 were capable of inhibiting both biofilm and filamentation. We found a reduction of 2.59 and 5.98 log (CFU/mL) of mature biofilms treated with SM-F1 and SM-F2, respectively. These results were superior to those found in our previous study when unfractionated *S. mutans* UA159 culture filtrate was tested on *C. albicans* biofilms, resulting in a 1 log (CFU/mL) reduction (Barbosa et al., 2016). The data indicate that the extraction and fractionation methods used in this study were adequate to separate and concentrate active molecules with antifungal activity. The inhibition is encouraging considering inhibition was driven by active elements that were not yet fully purified. Thus, we are working with a diluted product. Further purification and higher concentrations may yet yield more significant reductions.

Although biofilm formation is an important virulence mechanism for *Candida* species, previous *S. mutans* antifungal studies were focused on evaluating their effects on germ tubes and hyphae formation. Jarosz et al. (2009) verified that filter sterilized spent medium of *S. mutans* UA159 reduced *C. albicans* germ tube formation from 52 to 17%. These effects were attributed to the *quorum sensing* molecule CSP since a *S. mutans* mutant unable to produce CSP exhibited reduced germ tube inhibition compared to the wild-type strain. Joyner et al. (2010) also found a reduction in the morphological transition of *C. albicans* associated to the mutanobactin peptide produced by *S. mutans*. In co-cultures with mutanobactin-competent *S. mutans* strain, *C. albicans* grew only in the yeast form, whereas the co-culture with a mutanobactin deletion strain permitted *C. albicans* growth in a mycelium pattern. Vilchez et al. (2010) verified inhibitory effects of *S. mutans* extracts on *C. albicans* filamentation that were associated with fatty acid signaling molecule *trans*-2-decenoic acid. These authors observed that extracts from culture filtrates of *Streptococcus mitis*, *Streptococcus oralis*, and *Streptococcus sanguinis* also suppressed hyphae formation, but the strongest inhibition was achieved by the *S. mutans* extract.

In the present study, the *S. mutans* extract was fractionated to identify antifungal containing compounds. We found a significant reduction of hyphae formation when *C. albicans* were grown in contact with the SM-F2 fraction. In this study, *C. albicans* hyphae inhibition was not a condition of pH alteration since it was not a significant variant when the active fraction was added to the culture. The data reinforce that hyphae inhibition resulted from the presence of metabolites within the SM-F2 fraction. In addition, these metabolites seem to have strong inhibitory activity since the hyphae formation was inhibited in the presence of mammalian serum used in our filamentation assays. Mammalian serum is considered the strongest inducing factor among the conditions that stimulate *C. albicans* filamentation (Graham et al., 2017).

To complement the study about the inhibitory effects of the SM-F2 fraction on filamentation, we analyzed the expression of *C. albicans* genes when cells were cultured in serum bovine at 37°C and 5% CO_2_. We analyzed the expression of regulatory genes (*CPH1*, *EFG1*, and *UME6)* that integrate and respond to the environmental signals controlling the morphogenesis of *C. albicans* (Nobile and Mitchell, 2006; Lee et al., 2018). All these transcription factors (TFs) genes that contribute to the activation of the hyphal transcriptional program were downregulated by the SM-F2 fraction. Among them, *UME6* is a common downstream target of regulators promoting hyphal development and plays a role in the expression of *HWP1*, *ECE1*, *ALS3*, and *HGC1* genes (Nobile and Mitchell, 2006; Lee et al., 2018). Interestingly, SM-F2 provoked a 111.1- and 23.25-fold decreased in expression of the *UME6* and *HWP1* genes, respectively, under the same environmental conditions. On the other hand, the *YWP1* gene was upregulated with SM-F2 treatment. *YWP1* (Yeast Wall protein 1) is an anchored glycoprotein of the cell wall present only in the yeast form, absent in filamentous form and chlamydospores (Granger, 2012; McCall et al., 2019). Therefore, these results suggest that the activity of SM-F2 against filamentation observed in the microscopic analysis can be associated with the level of transcription in *C. albicans* cells that leads to retention in the yeast state.

Based on these promising data, we interrogated our *S*. *mutans* extracts using two different *in vivo* models. The *G. mellonella* model is a prompt and reliable method to evaluate toxicity and efficacy of antifungal agents (Junqueira and Mylonakis, 2019; Lin et al., 2019; Rossoni et al., 2019). The *S. mutans* extract tested concentrations were not toxic to the host. When the efficacy of SM-F1 and SM-F2 fractions were tested on candidiasis, only the SM-F2 fraction was able to prolong larvae survival. Treatment with SM-F2 increased the survival rate by 70% 24 h post-infection. These data were superior to those found in our previous study (Barbosa et al., 2016) in which the larvae were treated only with the culture filtrate of *S. mutans* UA159, resulting in an increase survival rate of 50% at 24 h post infection, suggesting the applied degree of purification increased potency. Therefore, the *G. mellonella* model provided evidences that the bioactivity of antifungal agents within SM-F2 were maintained under *in vivo* conditions.

Next, we used a well-established mouse model (Junqueira et al., 2005; Costa et al., 2013b; de Campos Rasteiro et al., 2014; Rossoni et al., 2015; Ribeiro et al., 2020) to evaluate the effects of the SM-F1 and SM-F2 fractions on oral candidiasis. Treatments with SM-F1 and SM-F2 reduced *C. albicans* cells in 0.79 and 0.91 Log (UFC/mL), respectively, compared to an untreated control group. In additional to affecting fungal oral colonization, the supernatant fractions were capable of inhibiting formation of pseudomembranous lesions on the dorsum tongue. The clinical observations were confirmed in the microscopic analysis, in which the SM-F1 and SM-F2 decreased the hyphae invasion, epithelial lesions and inflammatory infiltrate. Notably, the reductions observed were more pronounced by the treatment with SM-F2 than SM-F1. These data are very promising since the high efficacy of SM-F2 was confirmed in a dynamic infection model that mimics the oral candidiasis in humans. To our knowledge, this is the first report describing the effects of *S. mutans* culture extracts filtrate or mutanobactins on oral candidiasis model. Graham et al. (2017) investigated the development of oral candidiasis in mice treated with the EntV bacteriocin secreted by *Enterococcus faecalis*, a Gram-positive bacterium also presented in the oral microbiome. EntV reduced the fungal burden, hyphae invasion and inflammation in relation to non-treated group. Similarly, to the SM-F2 fraction used in our study, EntV did not eliminate the fungal colonization, but the hyphae invasion in the epithelial tissue was almost entirely eliminated (Graham et al., 2017).

Using GC-MS analysis, we identified compounds in the SM-F1 fraction that differed from the BHI control: Propanoic acid, 2,3-bis[(trimethylsilyl) oxy]-, trimethylsilyl ester; (3R)-3-methyl-1,4-bis(trimethylsilyl)piperazine-2,5-dione; pyrimidine, 5-methyl-2,4-bis[(trimethylsilyl)oxy]; and glucose, 2,3,4,5,6-pentakis-O-(trimethylsilyl). The compounds identified exclusively in the SM-F2 fraction were the nicotinic acid-TMS and Tryptophan, 2TMS. Some of these compounds are previously described in the literature to show antimicrobial activity. The propanoic acid (also called propionic acid) produced by *Propionibacterium acnes*, a commensal bacterium of human skin, was able to inhibit the growth of methicillin-resistant *Staphylococcus aureus*, *Escherichia coli*, and *C. albicans* (Wang et al., 2014). Nicotinic acid, also known as niacin or vitamin B3, is a water-soluble substance that has an important role in the human body, also its activity against *Staphylococcus aureus* was already demonstrated (Daglia et al., 1994). Nicotinamida has demonstrated antimicrobial activity against *C. albicans* (Pfaller et al., 2010), *Aspergillus* spp. (Dagenais and Keller, 2009) and *Botrytis cinerea* (Wang et al., 2019). Tryptophan-rich peptides have potent known antimicrobial activity attributed to the biochemical properties that facilitate crossing the microbial membranes without compromising their integrity, acting internally on nucleic acids and enzymes (Shagaghi et al., 2016; Mishra et al., 2018). Besides these compounds, many others can be present in the SM-F1 and SM-F2 fractions, however we were unable to identify them since a large number of substances from microbial extracts have not yet been described and inserted in the databases. According to Joyner et al. (2010), despite a variety of compounds such as peptides, lipids and acyl-homoserine lactones have emerged as communication agents among microorganisms, important contributions of many other secondary metabolites remains overlooked.

In addition, the compounds produced by microorganisms depend on environmental conditions; therefore, its identification can be limited by the study methods employed. In this study, the SM-F1 and SM-F2 fractions were obtained from pure cultures of *S. mutans*. Indeed, *quorum sensing* molecules have often been studied from pure laboratory cultures (Papenfort and Bassler, 2016; Mukherjee and Bassler, 2019), contributing to our understanding about the molecular mechanisms evolved in different microbial species (Mukherjee and Bassler, 2019). However, the uniform growth and the absence of mixed microbial communities can influence the functions of *quorum sensing* (Papenfort and Bassler, 2016). In this context, co-cultivation became an interesting tool to study *quorum sensing* molecules, in which co-cultures of two or more different microorganisms can mimic the ecological interactions (Marmann et al., 2014). The competition or antagonism experienced during co-cultivation may lead to an enhanced production of diversified compounds that are not detected in pure cultures (Marmann et al., 2014). Another important aspect that should be considered in future studies is the extension of antimicrobial activity of SM-F1 and SM-F2 for other microorganisms. It is probable that the antifungal effects of these fractions can be extended for other *Candida* species. Since *Candida glabrata*, *Candida tropicalis*, and *Candida krusei* also have an important role in the oral candidiasis, SM-F1 and SM-F2 fractions need to be tested on these non-*albicans Candida* species (Junqueira et al., 2011). Furthermore, the effects of extract and fractions of *S. mutans* can be explored against oral bacteria and dental biofilms, seeking mechanisms of action that may contribute for the control of oral bacterial infections, such as dental caries and periodontal diseases.

In conclusion, SM-F2 was the most effective fraction we tested with strong activity against *C. albicans* biofilms and filamentation, resulting in inhibition of candidiasis in an animal model. With further refinement, the extract demonstrates potential to be explored as an antifungal agent to treat oral candidiasis.

## Data Availability Statement

The raw data supporting the conclusions of this article will be made available by the authors, without undue reservation.

## Ethics Statement

The animal study was reviewed and approved by Ethics Committee on the Use of Animals of the ICT/UNESP under protocol 005/2016-CEUA- ICT-UNESP.

## Author Contributions

JJ and DS conceived and designed the experiments. JS, LF, and MA performed the experiments. RM contributed to preparation of *S. mutans* fractions and analysis by gas chromatography coupled to mass spectrometry. PB contributed to the gene expressions analysis. FR contributed to the experiments in oral candidiasis model. BF and EM contributed with reagents, materials or analysis tools in RIH/Brown University. JS, LF, RM, LS, and JJ analyzed the data. JS, LF, LS, BF, EM, and JJ wrote and revised the manuscript. All authors contributed to the article and approved the submitted version.

## Conflict of Interest

JJ, DS, JS, LF, and RM have obtained a patent for “Method of obtaining the extract and fractions of the supernatant of *S. mutans* and use of the extract and fractions of the supernatant *S. mutans* as antifungal” [patent number Br 10 2019 019528 2]. The remaining authors declare that the research was conducted in the absence of any commercial or financial relationships that could be construed as a potential conflict of interest.

## Supplementary Material

FIGURE S1 Validation of primers for qPCR. **(A)** Standard curve of the *HWP1* primer for the calculation of efficiency. **(B)** Specificity of the primers demonstrated by melting curve of *HWP1* gene obtained from *C. albicans* group.

FIGURE S2 Selection of the best reference gene (*ACT1, PMA1*, and *RPP2B*) using BestKeeper, NormFinder, Genorm, and Delta CT.

FIGURE S3 Light microscopy photomicrographs of *Candida* filamentation. Hyphae formation and presence of yeasts in the microscopic fields for the non-treated control group **(A,E)** and experimental groups treated with SM-F1 5 mg/mL **(B)**, SM-F1 10 mg/mL **(C)**, SM-F1 15 mg/mL **(D)**, SM-F2 5 mg/mL **(F)**, SM-F2 10 mg/mL **(G)**, and SM-F2 15 mg/mL **(H)**.

FIGURE S4 Measured pH in the *in vitro* filamentation assay. Median and SD of pH values in the medium for each well obtained in the following groups: non-treated control group, SM-F1 5 mg/mL, SM-F1 10 mg/mL, SM-F1 15 mg/mL, SM-F2 5 mg/mL, SM-F2 10 mg/mL, and SM-F2 15 mg/mL (H).

FIGURE S5 Chromatograms obtained by gas chromatography coupled to mass spectrometry (GC-MS). Comparisons between the SM-F1 fraction (red line) and control group of BHI media (black line). The black arrows indicate the peaks that are different between the SM-F1 and control. The blue arrows show the peaks of SM-F1 that indicate the identified compounds.

FIGURE S6 Chromatograms obtained by gas chromatography coupled to mass spectrometry (GC-MS). Comparisons between the SM-F2 fraction (pink) and control group of BHI media (black). The black arrows indicate the peaks that are different between the SM-F2 and control. The blue arrows show the peaks of SM-F2 that indicate the identified compounds.

## References

[B1] Barbosa J. O. Rossoni R. D. Vilela S. F. de Alvarenga J. A. Velloso Mdos S. Prata M. C. (2016). *Streptococcus mutans* can modulate biofilm formation and attenuate the virulence of *Candida albicans*. *PLoS One* 11:e0150457. 10.1371/journal.pone.0150457 26934196 PMC4774980

[B2] Becker M. R. Paster B. J. Leys E. J. Moeschberger M. L. Kenyon S. G. Galvin J. L. (2002). Molecular analysis of bacterial species associated with childhood caries. *J. Clin. Microbiol.* 40 1001–1009. 10.1128/jcm.40.3.1001-1009.2002 11880430 PMC120252

[B3] Bekal-Si Ali S. Hurtubise Y. Lavoie M. C. LaPointe G. (2002). Diversity of *Streptococcus mutans* bacteriocins as confirmed by DNA analysis using specific molecular probes. *Gene* 283 125–131. 10.1016/s0378-1119(01)00875-7 11867219

[B4] Berberi A. Noujeim Z. Aoun G. (2015). Epidemiology of oropharyngeal Candidiasis in human immunodeficiency virus/acquired immune deficiency syndrome patients and CD4+ counts. *J. Int. Oral Health* 7 20–23. PMC4385720 25878473

[B5] Breger J. Fuchs B. B. Aperis G. Moy T. I. Ausubel F. M. Mylonakis E. (2007). Antifungal chemical compounds identified using a *C. elegans* pathogenicity assay. *PLoS Pathog.* 3:e18. 10.1371/journal.ppat.0030018 17274686 PMC1790726

[B6] Chanda W. Joseph T. P. Wang W. Padhiar A. A. Zhong M. (2017). The potential management of oral candidiasis using anti-biofilm therapies. *Med. Hypotheses* 106 15–18. 10.1016/j.mehy.2017.06.029 28818264

[B7] Coronado-Castellote L. Jimenez-Soriano Y. (2013). Clinical and microbiological diagnosis of oral candidiasis. *J. Clin. Exp. Dent.* 5 e279–e286. 10.4317/jced.51242 24455095 PMC3892259

[B8] Costa A. C. Pereira C. A. Freire F. Junqueira J. C. Jorge A. O. (2013a). Methods for obtaining reliable and reproducible results in studies of Candida biofilms formed in vitro. *Mycoses* 56 614–622. 10.1111/myc.12092 23710618

[B9] Costa A. C. Pereira C. A. Junqueira J. C. Jorge A. O. (2013b). Recent mouse and rat methods for the study of experimental oral candidiasis. *Virulence* 4 391–399. 10.4161/viru.25199 23715031 PMC3714131

[B10] Dagenais T. R. Keller N. P. (2009). Pathogenesis of *Aspergillus fumigatus* in invasive Aspergillosis. *Clin. Microbiol. Rev.* 22 447–465. 10.1128/CMR.00055-08 19597008 PMC2708386

[B11] Daglia M. Cuzzoni M. T. Dacarro C. (1994). Antibacterial activity of coffee – relationship between biological-activity and chemical markers. *J. Agr. Food Chem.* 42 2273–2277. 10.1021/jf00046a036

[B12] de Barros P. P. Freire F. Rossoni R. D. Junqueira J. C. Jorge A. O. C. (2017). *Candida krusei* and *Candida glabrata* reduce the filamentation of *Candida albicans* by downregulating expression of HWP1 gene. *Folia Microbiol. (Praha)* 62 317–323. 10.1007/s12223-017-0500-4 28164244

[B13] de Campos Rasteiro V. M. da Costa A. C. Araujo C. F. de Barros P. P. Rossoni R. D. Anbinder A. L. (2014). Essential oil of *Melaleuca alternifolia* for the treatment of oral candidiasis induced in an immunosuppressed mouse model. *BMC Complement Altern. Med.* 14:489. 10.1186/1472-6882-14-489 25510285 PMC4301879

[B14] Falsetta M. L. Klein M. I. Colonne P. M. Scott-Anne K. Gregoire S. Pai C. H. (2014). Symbiotic relationship between *Streptococcus mutans* and *Candida albicans* synergizes virulence of plaque biofilms in vivo. *Infect. Immun.* 82 1968–1981. 10.1128/IAI.00087-14 24566629 PMC3993459

[B15] Garcia-Cuesta C. Sarrion-Perez M. G. Bagan J. V. (2014). Current treatment of oral candidiasis: a literature review. *J. Clin. Exp. Dent.* 6 e576–e582. 10.4317/jced.51798 25674329 PMC4312689

[B16] Graham C. E. Cruz M. R. Garsin D. A. Lorenz M. C. (2017). Enterococcus faecalis bacteriocin EntV inhibits hyphal morphogenesis, biofilm formation, and virulence of *Candida albicans*. *Proc. Natl. Acad. Sci. U.S.A.* 114 4507–4512. 10.1073/pnas.1620432114 28396417 PMC5410809

[B17] Granger B. L. (2012). Insight into the antiadhesive effect of yeast wall protein 1 of *Candida albicans*. *Eukaryot. Cell* 11 795–805. 10.1128/EC.00026-12 22505336 PMC3370456

[B18] Gulati M. Nobile C. J. (2016). *Candida albicans* biofilms: development, regulation, and molecular mechanisms. *Microbes Infect.* 18 310–321. 10.1016/j.micinf.2016.01.002 26806384 PMC4860025

[B19] Hnisz D. Bardet A. F. Nobile C. J. Petryshyn A. Glaser W. Schock U. (2012). A histone deacetylase adjusts transcription kinetics at coding sequences during *Candida albicans* morphogenesis. *PLoS Genet.* 8:e1003118. 10.1371/journal.pgen.1003118 23236295 PMC3516536

[B20] National Committee for Clinical Laboratory Standards (2002). Reference method for broth dilution antifungal susceptibility testing of yeasts. Approved standard, 2nd Edn, M27-A2 National Committee for Clinical Laboratory Standards, Wayne, Pa.

[B21] Jarosz L. M. Deng D. M. van der Mei H. C. Crielaard W. Krom B. P. (2009). *Streptococcus mutans* competence-stimulating peptide inhibits *Candida albicans* hypha formation. *Eukaryot. Cell* 8 1658–1664. 10.1128/EC.00070-09 19717744 PMC2772401

[B22] Joyner P. M. Liu J. Zhang Z. Merritt J. Qi F. Cichewicz R. H. (2010). Mutanobactin A from the human oral pathogen *Streptococcus mutans* is a cross-kingdom regulator of the yeast-mycelium transition. *Org. Biomol. Chem.* 8 5486–5489. 10.1039/c0ob00579g 20852771 PMC2992086

[B23] Junqueira J. C. Colombo C. E. Martins Jda S. Koga Ito C. Y. Carvalho Y. R. Jorge A. O. (2005). Experimental candidosis and recovery of *Candida albicans* from the oral cavity of ovariectomized rats. *Microbiol. Immunol.* 49 199–207. 10.1111/j.1348-0421.2005.tb03721.x 15781993

[B24] Junqueira J. C. Fuchs B. B. Muhammed M. Coleman J. J. Suleiman J. M. Vilela S. F. (2011). Oral *Candida albicans* isolates from HIV-positive individuals have similar in vitro biofilm-forming ability and pathogenicity as invasive Candida isolates. *BMC Microbiol.* 11:247. 10.1186/1471-2180-11-247 22053894 PMC3217868

[B25] Junqueira J. C. Mylonakis E. (2019). Current status and trends in alternative models to study fungal pathogens. *J. Fungi (Basel)* 5:12. 10.3390/jof5010012 30691083 PMC6463159

[B26] Kamiya R. U. Hofling J. F. Goncalves R. B. (2008). Frequency and expression of mutacin biosynthesis genes in isolates of *Streptococcus mutans* with different mutacin-producing phenotypes. *J. Med. Microbiol.* 57(Pt 5) 626–635. 10.1099/jmm.0.47749-0 18436597

[B27] Khan R. Rukke H. V. Hovik H. Amdal H. A. Chen T. Morrison D. A. (2016). Comprehensive transcriptome profiles of *Streptococcus mutans* UA159 map core streptococcal competence genes. *mSystems* 1:2. 10.1128/mSystems.00038-15 27822519 PMC5069739

[B28] Klein M. I. Bang S. Florio F. M. Hofling J. F. Goncalves R. B. Smith D. J. (2006). Genetic diversity of competence gene loci in clinical genotypes of *Streptococcus mutans*. *J. Clin. Microbiol.* 44 3015–3020. 10.1128/JCM.02024-05 16891531 PMC1594605

[B29] Lee J. H. Kim Y. G. Choi P. Ham J. Park J. G. Lee J. (2018). Antibiofilm and antivirulence activities of 6-Gingerol and 6-Shogaol against *Candida albicans* due to hyphal inhibition. *Front. Cell. Infect. Microbiol.* 8:299. 10.3389/fcimb.2018.00299 30211127 PMC6121036

[B30] Lin M. Y. Yuan Z. L. Hu D. D. Hu G. H. Zhang R. L. Zhong H. (2019). Effect of loureirin A against *Candida albicans* biofilms. *Chin. J. Nat. Med.* 17 616–623. 10.1016/S1875-5364(19)30064-0 31472899

[B31] Liu J. Y. Shi C. Wang Y. Li W. J. Zhao Y. Xiang M. J. (2015). Mechanisms of azole resistance in *Candida albicans* clinical isolates from Shanghai, China. *Res. Microbiol.* 166 153–161. 10.1016/j.resmic.2015.02.009 25748216

[B32] Livak K. J. Schmittgen T. D. (2001). Analysis of relative gene expression data using real-time quantitative PCR and the 2(-Delta Delta C(T)) Method. *Methods* 25 402–408. 10.1006/meth.2001.1262 11846609

[B33] Marmann A. Aly A. H. Lin W. Wang B. Proksch P. (2014). Co-cultivation–a powerful emerging tool for enhancing the chemical diversity of microorganisms. *Mar. Drugs* 12 1043–1065. 10.3390/md12021043 24549204 PMC3944530

[B34] Martins Jda S. Junqueira J. C. Faria R. L. Santiago N. F. Rossoni R. D. Colombo C. E. (2011). Antimicrobial photodynamic therapy in rat experimental candidiasis: evaluation of pathogenicity factors of *Candida albicans*. *Oral Surg. Oral Med. Oral Pathol. Oral Radiol. Endod.* 111 71–77. 10.1016/j.tripleo.2010.08.012 21176823

[B35] Matsubara V. H. Wang Y. Bandara H. M. Mayer M. P. Samaranayake L. P. (2016). Probiotic lactobacilli inhibit early stages of *Candida albicans* biofilm development by reducing their growth, cell adhesion, and filamentation. *Appl. Microbiol. Biotechnol.* 100 6415–6426. 10.1007/s00253-016-7527-3 27087525

[B36] McCall A. D. Pathirana R. U. Prabhakar A. Cullen P. J. Edgerton M. (2019). *Candida albicans* biofilm development is governed by cooperative attachment and adhesion maintenance proteins. *NPJ Biofilms Microb.* 5:21. 10.1038/s41522-019-0094-5 31452924 PMC6707306

[B37] Medina R. P. Araujo A. R. Batista J. M. Cardoso C. L. Seidl C. Vilela A. F. L. (2019). Botryane terpenoids produced by *Nemania bipapillata*, an endophytic fungus isolated from red alga *Asparagopsis taxiformis* – *Falkenbergia* stage. *Sci. Rep.* 9:12318. 10.1038/s41598-019-48655-7 31444403 PMC6707159

[B38] Mishra A. K. Choi J. Moon E. Baek K. H. (2018). Tryptophan-rich and proline-rich antimicrobial peptides. *Molecules* 23:815. 10.3390/molecules23040815 29614844 PMC6017362

[B39] Mitchell T. J. (2003). The pathogenesis of streptococcal infections: from tooth decay to meningitis. *Nat. Rev. Microbiol.* 1 219–230. 10.1038/nrmicro771 15035026

[B40] Moges B. Bitew A. Shewaamare A. (2016). Spectrum and the *in vitro* antifungal susceptibility pattern of yeast isolates in ethiopian HIV patients with oropharyngeal candidiasis. *Int. J. Microbiol.* 2016:3037817. 10.1155/2016/3037817 26880925 PMC4736391

[B41] Mukherjee S. Bassler B. L. (2019). Bacterial quorum sensing in complex and dynamically changing environments. *Nat. Rev. Microbiol.* 17 371–382. 10.1038/s41579-019-0186-5 30944413 PMC6615036

[B42] Nailis H. Coenye T. Van Nieuwerburgh F. Deforce D. Nelis H. J. (2006). Development and evaluation of different normalization strategies for gene expression studies in *Candida albicans* biofilms by real-time PCR. *BMC Mol. Biol.* 7:25. 10.1186/1471-2199-7-25 16889665 PMC1557526

[B43] Nobile C. J. Mitchell A. P. (2006). Genetics and genomics of *Candida albicans* biofilm formation. *Cell Microbiol.* 8 1382–1391. 10.1111/j.1462-5822.2006.00761.x 16848788

[B44] Papenfort K. Bassler B. L. (2016). Quorum sensing signal-response systems in Gram-negative bacteria. *Nat. Rev. Microbiol.* 14 576–588. 10.1038/nrmicro.2016.89 27510864 PMC5056591

[B45] Patton L. L. (2016). Current strategies for prevention of oral manifestations of human immunodeficiency virus. *Oral Surg. Oral Med. Oral Pathol. Oral Radiol.* 121 29–38. 10.1016/j.oooo.2015.09.004 26679357

[B46] Peleg A. Y. Hogan D. A. Mylonakis E. (2010). Medically important bacterial-fungal interactions. *Nat. Rev. Microbiol.* 8 340–349. 10.1038/nrmicro2313 20348933

[B47] Pellati F. Benvenuti S. (2007). Chromatographic and electrophoretic methods for the analysis of phenethylamine [corrected] alkaloids in *Citrus aurantium*. *J. Chromatogr. A* 1161 71–88. 10.1016/j.chroma.2007.05.097 17582424

[B48] Pereira-Cenci T. Deng D. M. Kraneveld E. A. Manders E. M. Del Bel Cury A. A. Ten Cate J. M. (2008). The effect of *Streptococcus mutans* and *Candida glabrata* on *Candida albicans* biofilms formed on different surfaces. *Arch. Oral Biol.* 53 755–764. 10.1016/j.archoralbio.2008.02.015 18395698

[B49] Pfaller M. A. Diekema D. J. Gibbs D. L. Newell V. A. Ellis D. Tullio V. (2010). Results from the ARTEMIS DISK global antifungal surveillance study, 1997 to 2007: a 10.5-year analysis of susceptibilities of *Candida* species to fluconazole and voriconazole as determined by CLSI standardized disk diffusion. *J. Clin. Microbiol.* 48 1366–1367. 10.1128/JCM.02117-09 20164282 PMC2849609

[B50] Ribeiro F. C. Junqueira J. C. Dos Santos J. D. de Barros P. P. Rossoni R. D. Shukla S. (2020). Development of probiotic formulations for oral candidiasis prevention: gellan gum as a carrier to deliver *Lactobacillus paracasei* 28.4. *Antimicrob. Agents Chemother.* 64:e02323-19. 10.1128/AAC.02323-19 32253208 PMC7269487

[B51] Rossoni R. D. Barbosa J. O. Vilela S. F. dos Santos J. D. de Barros P. P. Prata M. C. (2015). Competitive interactions between *C. albicans*, *C. glabrata* and *C. krusei* during biofilm formation and development of experimental candidiasis. *PLoS One* 10:e0131700. 10.1371/journal.pone.0131700 26146832 PMC4493022

[B52] Rossoni R. D. de Barros P. P. de Alvarenga J. A. Ribeiro F. C. Velloso M. D. S. Fuchs B. B. (2018). Antifungal activity of clinical *Lactobacillus* strains against *Candida albicans* biofilms: identification of potential probiotic candidates to prevent oral candidiasis. *Biofouling* 34 212–225. 10.1080/08927014.2018.1425402 29380647

[B53] Rossoni R. D. Fuchs B. B. de Barros P. P. Velloso M. D. Jorge A. O. Junqueira J. C. (2017). *Lactobacillus paracasei* modulates the immune system of *Galleria mellonella* and protects against *Candida albicans* infection. *PLoS One* 12:e0173332. 10.1371/journal.pone.0173332 28267809 PMC5340386

[B54] Rossoni R. D. Ribeiro F. C. Dos Santos H. F. S. Dos Santos J. D. Oliveira N. S. Dutra M. (2019). Galleria mellonella as an experimental model to study human oral pathogens. *Arch. Oral Biol.* 101 13–22. 10.1016/j.archoralbio.2019.03.002 30856377

[B55] Salvatori O. Puri S. Tati S. Edgerton M. (2016). Innate immunity and saliva in *Candida albicans*-mediated oral diseases. *J. Dent. Res.* 95 365–371. 10.1177/0022034515625222 26747422 PMC4802782

[B56] Seneviratne C. J. Rosa E. A. (2016). Editorial: antifungal drug discovery: new theories and new therapies. *Front. Microbiol.* 7:728. 10.3389/fmicb.2016.00728 27242745 PMC4876608

[B57] Shagaghi N. Palombo E. A. Clayton A. H. Bhave M. (2016). Archetypal tryptophan-rich antimicrobial peptides: properties and applications. *World J. Microbiol. Biotechnol.* 32:31. 10.1007/s11274-015-1986-z 26748808

[B58] Sherry L. Rajendran R. Lappin D. F. Borghi E. Perdoni F. Falleni M. (2014). Biofilms formed by *Candida albicans* bloodstream isolates display phenotypic and transcriptional heterogeneity that are associated with resistance and pathogenicity. *BMC Microbiol.* 14:182. 10.1186/1471-2180-14-182 24996549 PMC4105547

[B59] Shu C. Sun L. Zhang W. (2016). Thymol has antifungal activity against *Candida albicans* during infection and maintains the innate immune response required for function of the p38 MAPK signaling pathway in *Caenorhabditis elegans*. *Immunol. Res.* 64 1013–1024. 10.1007/s12026-016-8785-y 26783030

[B60] Takakura N. Sato Y. Ishibashi H. Oshima H. Uchida K. Yamaguchi H. (2003). A novel murine model of oral candidiasis with local symptoms characteristic of oral thrush. *Microbiol. Immunol.* 47 321–326. 10.1111/j.1348-0421.2003.tb03403.x 12825893

[B61] Thein Z. M. Samaranayake Y. H. Samaranayake L. P. (2006). Effect of oral bacteria on growth and survival of *Candida albicans* biofilms. *Arch. Oral Biol.* 51 672–680. 10.1016/j.archoralbio.2006.02.005 16620775

[B62] Vazquez J. A. (2010). Optimal management of oropharyngeal and esophageal candidiasis in patients living with HIV infection. *HIV AIDS (Auckl)* 2 89–101. 22096388 10.2147/hiv.s6660 PMC3218701

[B63] Venkatasalu M. R. Murang Z. R. Ramasamy D. T. R. Dhaliwal J. S. (2020). Oral health problems among palliative and terminally ill patients: an integrated systematic review. *BMC Oral Health* 20:79. 10.1186/s12903-020-01075-w 32188452 PMC7079519

[B64] Vilchez R. Lemme A. Ballhausen B. Thiel V. Schulz S. Jansen R. (2010). *Streptococcus mutans* inhibits *Candida albicans* hyphal formation by the fatty acid signaling molecule trans-2-decenoic acid (SDSF). *Chembiochem* 11 1552–1562. 10.1002/cbic.201000086 20572249

[B65] Vilela S. F. Barbosa J. O. Rossoni R. D. Santos J. D. Prata M. C. Anbinder A. L. (2015). *Lactobacillus acidophilus* ATCC 4356 inhibits biofilm formation by *C. albicans* and attenuates the experimental candidiasis in *Galleria mellonella*. *Virulence* 6 29–39. 10.4161/21505594.2014.981486 25654408 PMC4603435

[B66] Wang G. Cui P. Bai H. Wei S. Li S. (2019). Late-stage C-H functionalization of nicotinamides for the expedient discovery of novel antifungal leads. *J. Agric. Food Chem.* 67 11901–11910. 10.1021/acs.jafc.9b05349 31584275

[B67] Wang Y. Dai A. Huang S. Kuo S. Shu M. Tapia C. P. (2014). Propionic acid and its esterified derivative suppress the growth of methicillin-resistant *Staphylococcus aureus* USA300. *Benef. Microbes* 5 161–168. 10.3920/BM2013.0031 24686580

[B68] Zhang K. Ou M. Wang W. Ling J. (2009). Effects of quorum sensing on cell viability in *Streptococcus mutans* biofilm formation. *Biochem. Biophys. Res. Commun.* 379 933–938. 10.1016/j.bbrc.2008.12.175 19138664

[B69] Zhang L. W. Fu J. Y. Hua H. Yan Z. M. (2016). Efficacy and safety of miconazole for oral candidiasis: a systematic review and meta-analysis. *Oral Dis.* 22 185–195. 10.1111/odi.12380 26456226

