# Supplementary figures and images for: Streptococcus mutans Secreted Products Inhibit Candida albicans Induced Oral Candidiasis
Source: Front Microbiol. 2020 Jul 15;11:1605. doi: 10.3389/fmicb.2020.01605 (PMC7374982; doi:10.3389/fmicb.2020.01605)

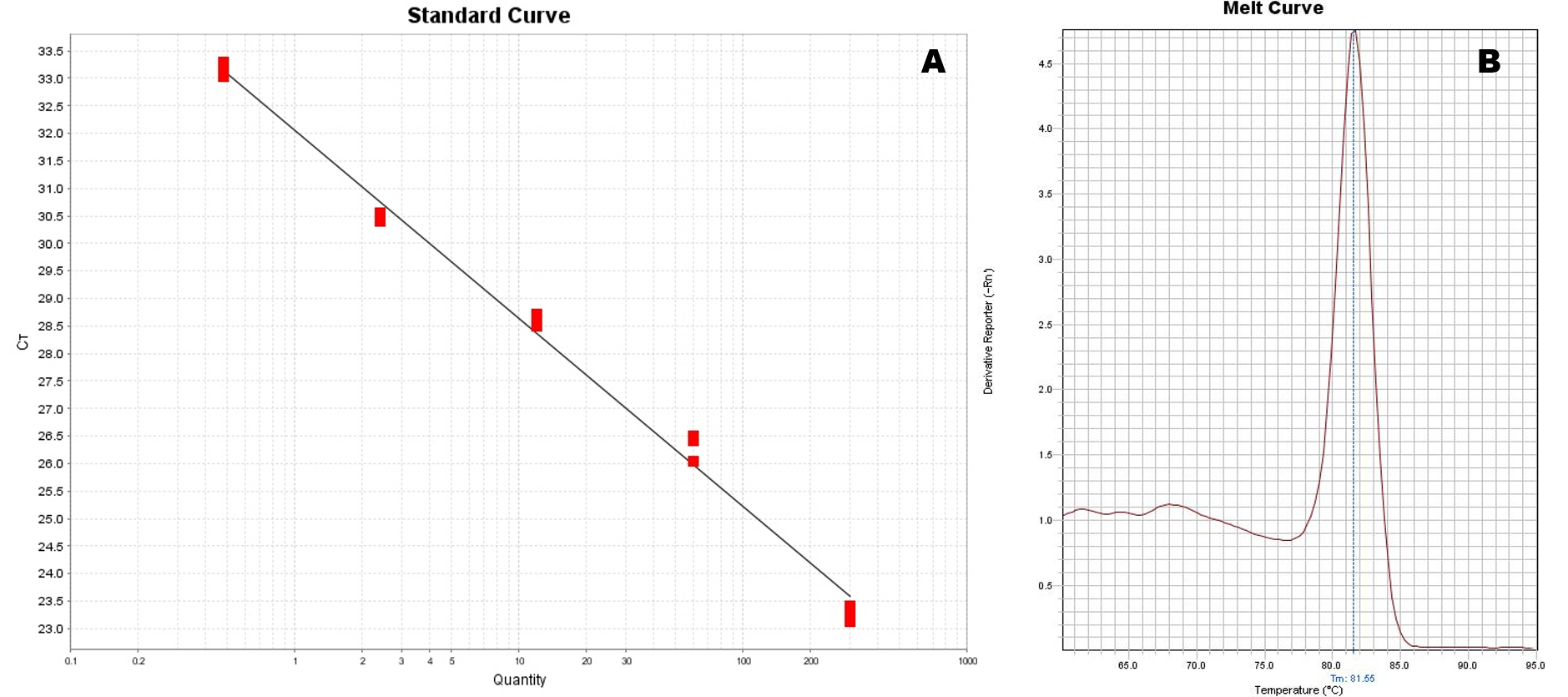

Supplement: FIGURE S1 — Validation of primers for qPCR. (A) Standard curve of the HWP1 primer for the calculation of efficiency. (B) Specificity of the primers demonstrated by melting curve of HWP1 gene obtained from C. albicans group. [file Image_1.PNG]

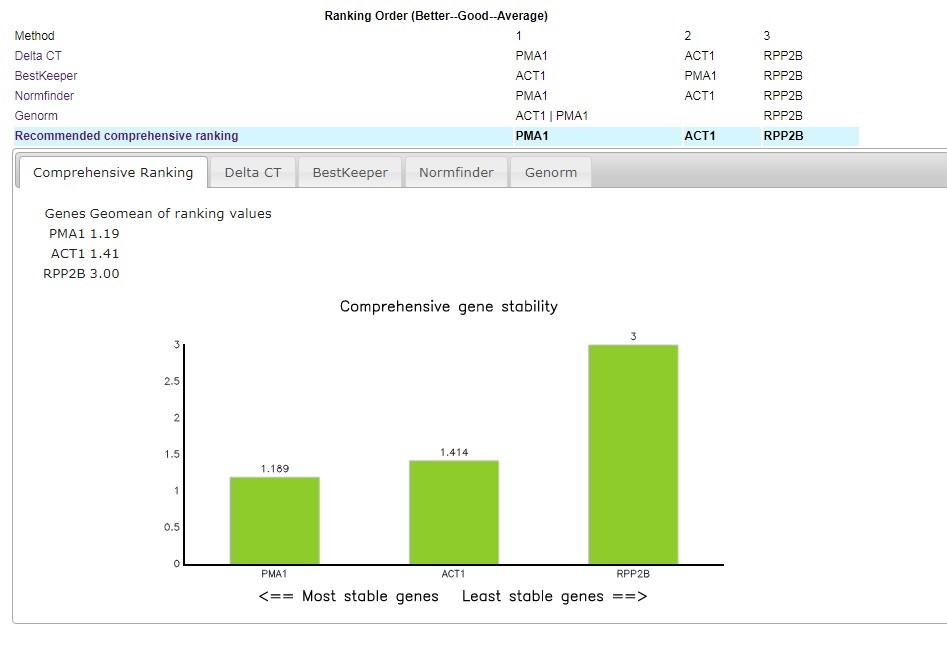

Supplement: FIGURE S2 — Selection of the best reference gene (ACT1, PMA1, and RPP2B) using BestKeeper, NormFinder, Genorm, and Delta CT. [file Image_2.JPEG]

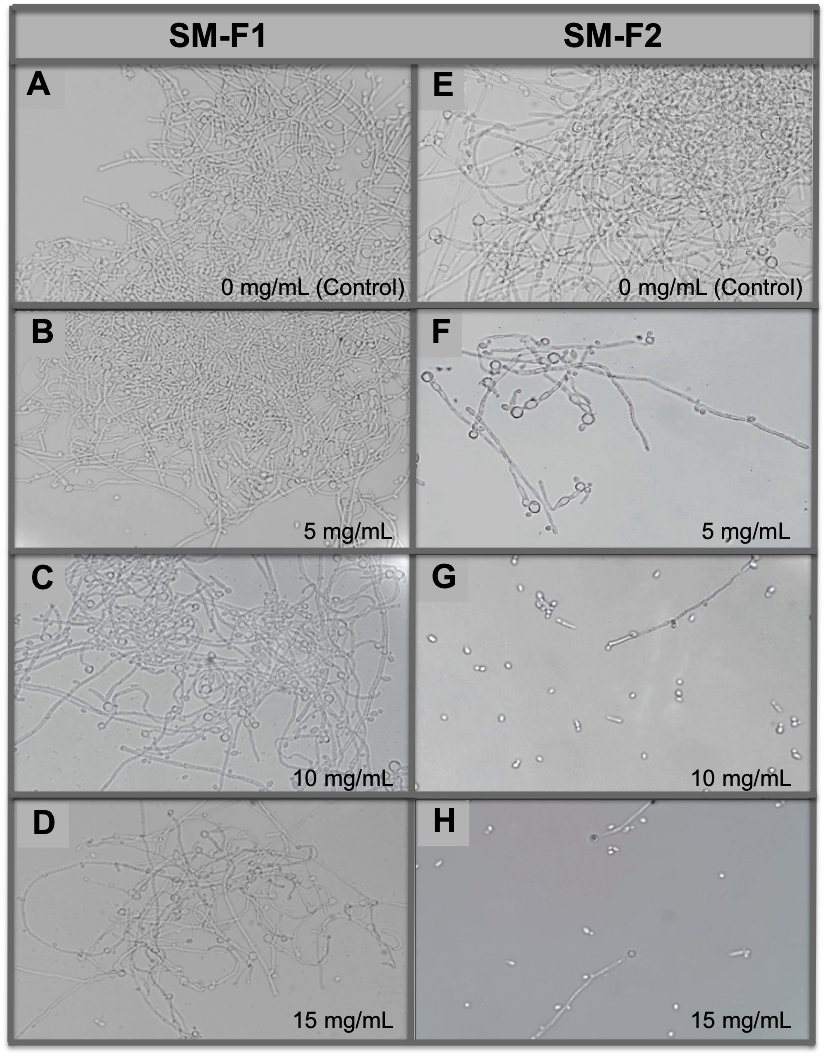

Supplement: FIGURE S3 — Light microscopy photomicrographs of Candida filamentation. Hyphae formation and presence of yeasts in the microscopic fields for the non-treated control group (A,E) and experimental groups treated with SM-F1 5 mg/mL (B), SM-F1 10 mg/mL (C), SM-F1 15 mg/mL (D), SM-F2 5 mg/mL (F), SM-F2 10 mg/mL (G), and SM-F2 15 mg/mL (H). [file Image_3.PNG]

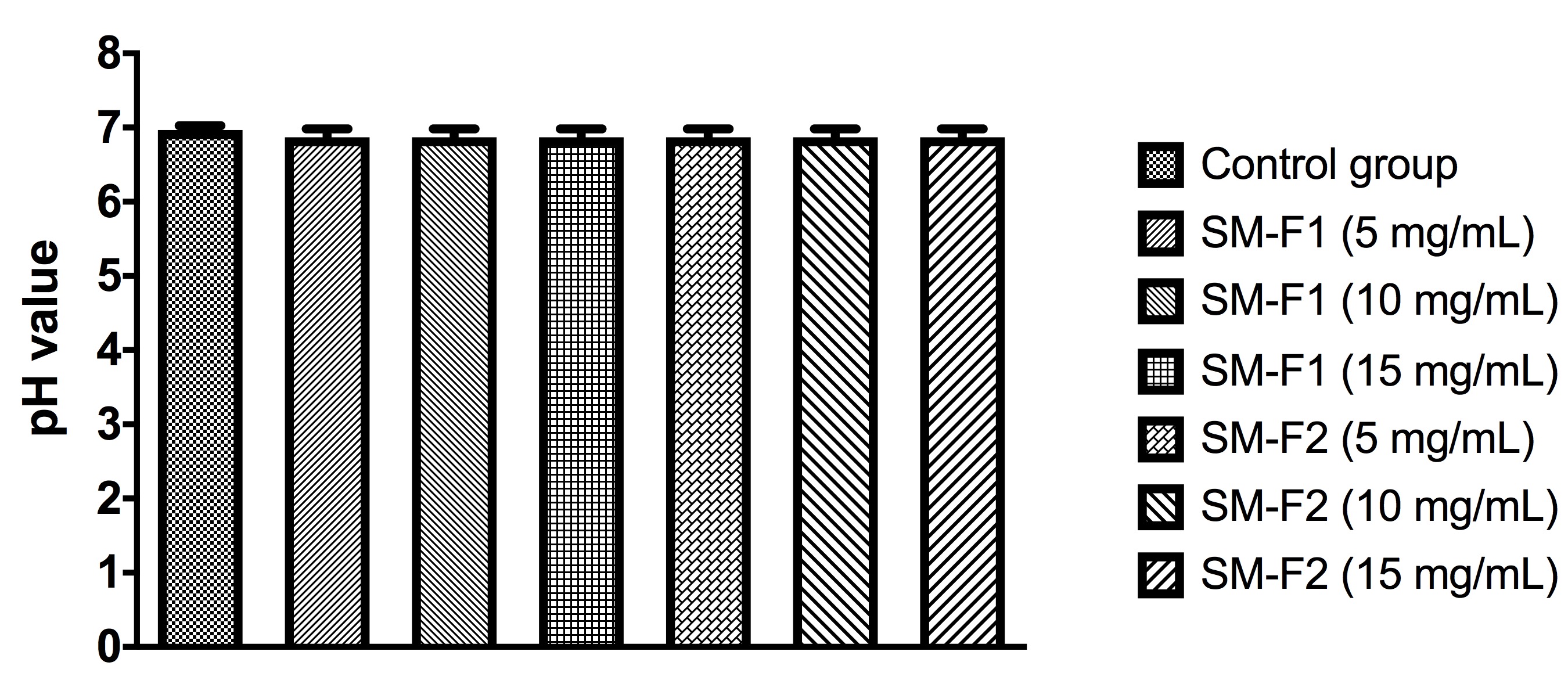

Supplement: FIGURE S4 — Measured pH in the in vitro filamentation assay. Median and SD of pH values in the medium for each well obtained in the following groups: non-treated control group, SM-F1 5 mg/mL, SM-F1 10 mg/mL, SM-F1 15 mg/mL, SM-F2 5 mg/mL, SM-F2 10 mg/mL, and SM-F2 15 mg/mL (H). [file Image_4.JPEG]

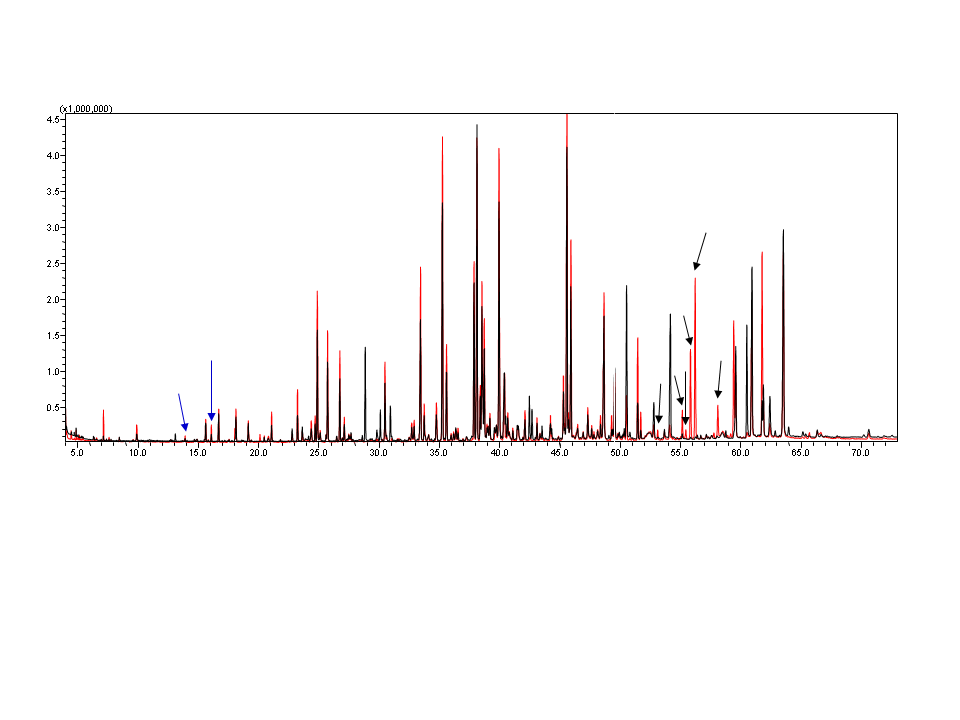

Supplement: FIGURE S5 — Chromatograms obtained by gas chromatography coupled to mass spectrometry (GC-MS). Comparisons between the SM-F1 fraction (red line) and control group of BHI media (black line). The black arrows indicate the peaks that are different between the SM-F1 and control. The blue arrows show the peaks of SM-F1 that indicate the identified compounds. [file Image_5.TIFF]
